# Information spreading by a combination of MEG source estimation and multivariate pattern classification

**DOI:** 10.1371/journal.pone.0198806

**Published:** 2018-06-18

**Authors:** Masashi Sato, Okito Yamashita, Masa-aki Sato, Yoichi Miyawaki

**Affiliations:** 1 Graduate School of Informatics and Engineering, The University of Electro-Communications, Tokyo, Japan; 2 Japan Society for the Promotion of Science, Tokyo, Japan; 3 Neural Information Analysis Laboratories, Advanced Telecommunications Research Institute International, Kyoto, Japan; 4 Brain Functional Imaging Technologies Group, CiNet, Osaka, Japan; 5 Center for Advanced Intelligence Project, RIKEN, Tokyo, Japan; 6 JST, PRESTO, Tokyo, Japan; Boston Children's Hospital / Harvard Medical School, UNITED STATES

## Abstract

To understand information representation in human brain activity, it is important to investigate its fine spatial patterns at high temporal resolution. One possible approach is to use source estimation of magnetoencephalography (MEG) signals. Previous studies have mainly quantified accuracy of this technique according to positional deviations and dispersion of estimated sources, but it remains unclear how accurately MEG source estimation restores information content represented by spatial patterns of brain activity. In this study, using simulated MEG signals representing artificial experimental conditions, we performed MEG source estimation and multivariate pattern analysis to examine whether MEG source estimation can restore information content represented by patterns of cortical current in source brain areas. Classification analysis revealed that the corresponding artificial experimental conditions were predicted accurately from patterns of cortical current estimated in the source brain areas. However, accurate predictions were also possible from brain areas whose original sources were not defined. Searchlight decoding further revealed that this unexpected prediction was possible across wide brain areas beyond the original source locations, indicating that information contained in the original sources can spread through MEG source estimation. This phenomenon of “information spreading” may easily lead to false-positive interpretations when MEG source estimation and classification analysis are combined to identify brain areas that represent target information. Real MEG data analyses also showed that presented stimuli were able to be predicted in the higher visual cortex at the same latency as in the primary visual cortex, also suggesting that information spreading took place. These results indicate that careful inspection is necessary to avoid false-positive interpretations when MEG source estimation and multivariate pattern analysis are combined.

## Introduction

Close investigation of human brain activity patterns plays a vital role in revealing how information is represented in the human brain. However, no method can directly measure fine spatial patterns of human brain activity at a high temporal resolution. Functional magnetic resonance imaging (fMRI) can capture the spatial patterns of human brain activity, but it lacks temporal resolution, as it measures metabolic processes reflected by blood-oxygenation-level-dependent signals. In contrast, magnetoencephalography (MEG) and electroencephalography (EEG) can capture the fast dynamics of brain activity patterns via electromagnetic signals that propagate from neurons; however, both of these techniques lack fine spatial resolution because their signals are measured by a small number of sensors placed around the head.

Given these constraints, researchers have tried to extract necessary information by solving an inverse problem to estimate cortical current source from measured MEG/EEG signals. MEG is more suitable than EEG for this purpose because of the homogeneous magnetic permeability of brain tissues. The typical approach for MEG source estimation uses an equivalent current dipole or distributed source model, assuming a few or many (typically 10^3–4^) current dipoles in the brain. Although additional constraints are necessary to resolve the ill-posed condition, a distributed source model seems preferable to analyze fine spatial patterns of brain activity because of its descriptive power. However, no method can reconstruct cortical current source with perfect accuracy because of the ill-posed nature of MEG source estimation. It is hence important to be aware of limitations of MEG source estimation.

Previous studies have mainly evaluated the performance of MEG source estimation with regard to the accuracy of source position estimation. Thus, error has typically been quantified in terms of spatial displacement [1], dispersion [2–5], and overlap/non-overlap of the estimated sources relative to the original ones [6–8]. However, it remains unclear how accurately the spatial patterns of the original source are restored through MEG source estimation.

Restorability of the spatial patterns of the original sources through MEG source estimation is crucial for investigating the information content represented by brain activity patterns [9–17]. Previous fMRI studies have shown that multivariate pattern analysis (MVPA) allows information of images seen by participants to be extracted from the spatial patterns of their brain activity [18–20]. Recent MEG studies have also applied MVPA to estimated cortical current and evaluated the information represented by them [21–24]. This progress emphasizes the importance of evaluating whether the original spatial pattern of the source is preserved or whether a spurious pattern is fabricated through MEG source estimation.

**Fig 1:**
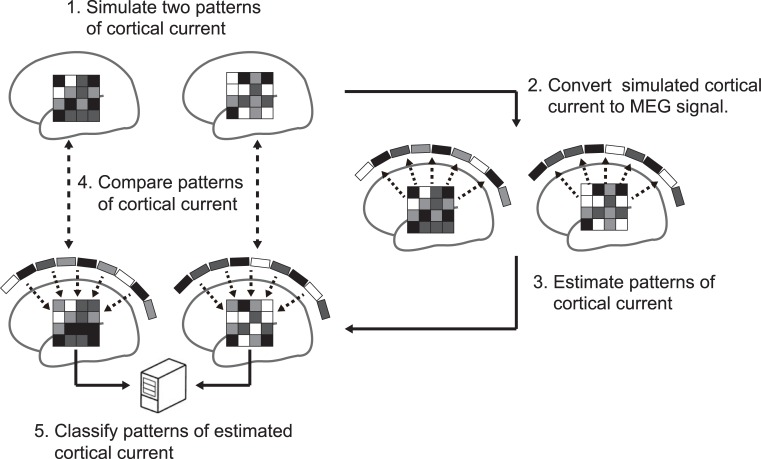
Schematic view of multivariate pattern analysis of cortical current estimated by MEG source estimation. Two patterns of source cortical current are simulated (step 1) and then converted to MEG signals (step 2). Next, cortical current is estimated from the converted MEG signals using MEG source estimation methods (step 3). To evaluate pattern reproducibility and information restorability, the original and estimated patterns of cortical current are compared using multivariate pattern analyses (steps 4 and 5).

In this study, we examine whether MEG source estimation can restore a pattern of the original cortical current and the represented information content of the source brain areas (Fig 1). For this purpose, we assumed patterns of source cortical current in certain areas on participants’ cortical surface models that would encode differences in artificial experimental conditions as multidimensional patterns. Given the source cortical current, we calculate the expected magnetic fields at the MEG sensors and estimate the spatial patterns of the cortical current from the simulated MEG sensor signals. We use four representative methods of MEG source estimation based on distributed source models. We quantify the similarity between the spatial patterns of the original and estimated cortical currents and show significant correlation between them. We then demonstrate that the estimated cortical current can be used to predict the corresponding artificial experimental conditions by pattern classification analysis but that significant prediction is possible even in cortical areas where the original source is not defined. This unexpected phenomenon makes it appear as if represented information spreads over a wide cortical area when MEG source estimation is used, which could lead to misinterpretations about the cortical areas that represent target information. Further analyses confirm that this information spreading can be similarly observed in real MEG data.

## Methods

### Modeling cortical surfaces

Magnetic resonance imaging (MRI) was used to obtain each participant’s cortical structural images. Cortical surface models were then extracted from the cortical structural images to simulate source cortical current. The simulation described in the following sections were conducted for each participant independently using the individual cortical surface models.

#### Participants

Cortical structural images were obtained from nine participants (eight male and one female). They participated in our study voluntarily, and the same participants also took part in the MEG experiments described later. Each participant gave written informed consent before participating. The procedure was approved by the institutional review board of The University of Electro-Communications and Advanced Telecommunications Research Institute International (ATR) Brain Activity Imaging Center.

#### MRI acquisition

Cortical structural images were obtained using 3.0-Tesla Siemens MAGNETOM Trio A Tim and Prisma fit scanners located at the ATR Brain Activity Imaging Center. T1-weighted magnetization-prepared rapid-acquisition gradient-echo (MP-RAGE) fine structural images of the whole head were acquired for each participant (208 sagittal slices; TR, 2250 ms; TE, 3.06 ms; TI, 900 ms; flip angle, 9°; field of view, 256 × 256 mm; voxel size, 1.0 × 1.0 × 1.0 mm; the same parameters were used on the Trio and Prisma scanners).

#### Extraction of cortical surfaces

The cortical surface was defined as a polygon model of the gray matter surface extracted from each participant’s MRI data using the FreeSurfer software suite (http://surfer.nmr.mgh.harvard.edu/), with about 300,000 vertices. The polygonized cortical surface models were then imported into Brainstorm [25], and the number of vertices was downsampled to 15,002 to reduce computational load.

### Simulation of source cortical current

**Fig 2:**
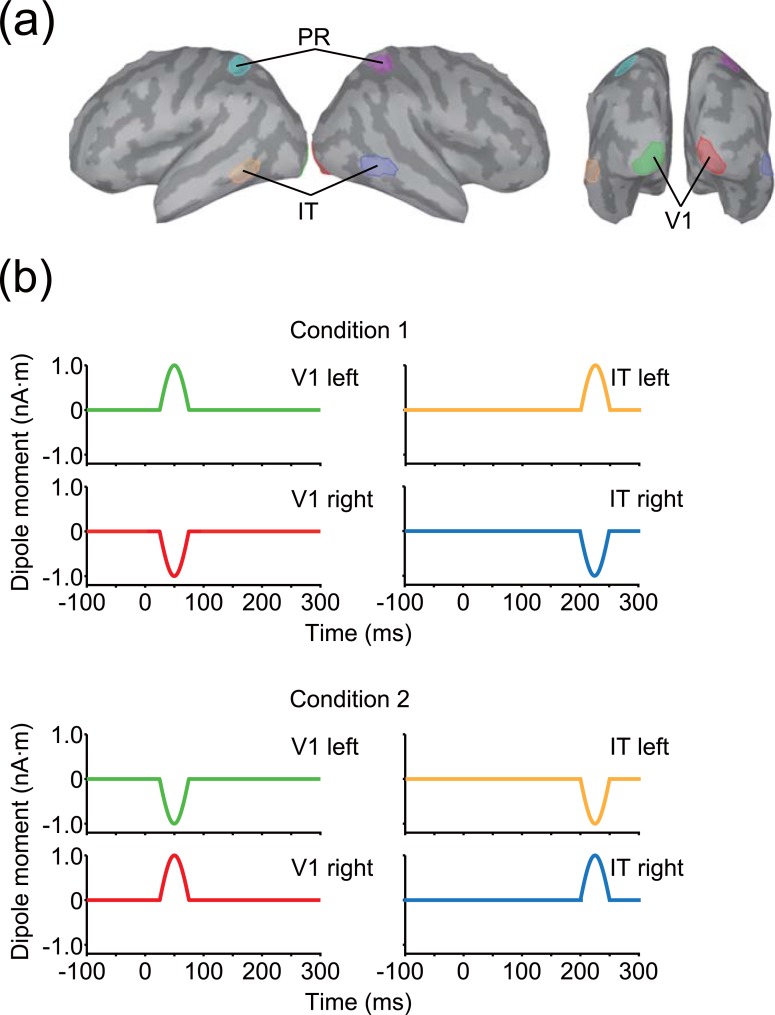
ROIs and patterns of source cortical current. (a) Three bilateral ROIs arranged on the cortical surface of the standard brain. (b) Source cortical current within each ROI. Line colors correspond to ROI colors shown in (a).

A source cortical current was simulated for each participant’s cortical surface model with a time course designed to roughly imitate the evoked response caused by visual stimulation along the visual cortical hierarchy. The time period of the source cortical current was defined during −100–300 ms, which corresponds to a single trial in the simulation. We assumed two source areas in different time windows and ROIs: at 25–75 ms in the primary visual cortex (V1) and at 200–250 ms in the inferotemporal cortex (IT; Fig 2). ROIs were manually defined on the ICBM152 standard brain [26] and then projected onto each participant’s cortical surface model using FreeSurfer’s spherical morphing procedure (Fig 2A). In addition to V1 and IT, the parietal cortex (PR) was also defined without any sources for control analyses (Fig 2A). V1 was defined to encompass the occipital pole, IT was defined to overlap with the inferoposterior part of the temporal lobe, and PR was defined as the middle part of lateral parietal lobe. Each ROI enclosed 120 vertices (60 vertices for each hemisphere). No sources were assumed on other vertices. The time origin (0 ms) was considered to be the onset of visual stimulation. Although we used this particular timing setup, it does not affect the presented results qualitatively unless the two source activity temporally overlap.

Each of the sources was a combination of multiple current dipoles with the same amplitude within each hemisphere, but different amplitudes across hemispheres. Current dipoles were placed at all vertices of the cortical surface whose directions were perpendicular to the cortical surface (the positive direction was defined as from the inside to the outside of the brain). Note that current dipoles with non-zero amplitude was placed only in the source ROIs whereas those placed in other areas had zero amplitude. The amplitude of the source cortical current in each ROI was temporally modulated as a sinusoidal waveform whose phase started at zero and ended at *π* within each time window (Fig 2B). Thus, the amplitude was maximal at the middle of each time window (50 ms and 225 ms for V1 and IT, respectively). We created two artificial experimental conditions with differences in maximum amplitude for each hemisphere: for condition 1, the maximum amplitude of the source cortical current was 1 nA⋅m for the left hemisphere and −1 nA⋅m for the right, while condition 2 was the opposite of condition 1 across hemispheres. Thus, the mean amplitude of the source cortical current across hemispheres was the same in both of the artificial experimental conditions.

### Calculation of MEG sensor signals

To simulate MEG sensor signals generated by the original cortical current, we measured head position relative to the MEG sensors for each participant.

#### Head position measurement and registration

We first measured each participant’s head shape and the positions of five electromagnetic marker coils attached to the participant’s head (three on the forehead and two on the ear tragi) using a three-dimensional digitizer (FastSCAN, Polhemus Inc., USA). The magnetic field generated by the marker coils was then measured by an MEG system (PQ1400RM, Yokogawa Electric Co., Japan) with 400 SQUID sensors (210 axial and 190 planar gradiometers), and each participant’s head position was estimated from the measured signals. Each participant’s head position in the MEG system was then coregistered to the cortical structural image of the same individual’s MRI data. The positional relationship between the MEG sensors and the cortical surface was determined from each participant’s coregistration results.

#### MEG signal synthesis

MEG sensor signals were calculated by a forward model that describes magnetic propagation processes from the original cortical current to the MEG sensors. The forward model can be described as

b=G·j+e (1)

where **b** (ℝ^*N*×1^ vector; *N*, number of MEG sensors) represents measured MEG signals, **G** (ℝ^*N*×*M*^ matrix; *M*, number of vertices on the cortical surface) represents a leadfield matrix, **j** (ℝ^*M*×1^ vector) represents the original cortical current, and **e** (ℝ^*N*×1^ vector) represents sensor noise. Each column of the leadfield matrix indicates the amplitude of the MEG signals generated by a unit current dipole of the corresponding vertex. **G** was calculated by the boundary element method using the OpenMEEG software package [27,28]. Sensor noise was modeled to follow a Gaussian distribution *N*(0,*σ*^2^). In this simulation, *σ* was set to twice the largest MEG signal amplitude across all time points and sensors to represent the low signal-to-noise ratio that would occur in an actual experiment. The noise was added to each time point and MEG sensor independently. We constructed five artificial experimental runs by repeating this procedure 50 times for each artificial experimental condition. Each run consisted of 100 trials in total.

### Estimation of cortical current from simulated MEG sensor signals

Suppose that $\hat{j}$ is an estimate value of **j** (ℝ^*M*×1^ vector) given **b** and **G**. To estimate $\hat{j}$, we need to solve an inverse problem that is ill-posed because there are fewer MEG sensors than assumed current dipoles on the cortical surface. To obtain a unique solution, we used the following four representative frameworks for MEG source estimation, which have been used extensively in recent studies.

#### L2-norm regularization

We first tested L2-norm regularization, whose objective function is written as

$$E\left(\hat{j}\right)={\left(b-G\cdot \hat{j}\right)}^{T}\cdot C^{-1}\cdot \left(b-G\cdot \hat{j}\right)+\lambda {\hat{j}}^{T}\cdot R^{-1}\cdot \hat{j}$$ (2)

where **C** is the ℝ^*N*×*N*^ noise covariance matrix, **R** is the ℝ^*M*×*M*^ source covariance matrix, *λ* is a regularization parameter, and T indicates the transpose. The first term on the right-hand side of Eq (2) indicates the estimation error weighted by noise covariance, and the second term indicates L2-norm regularization weighted by source covariance. This framework is known as minimum norm estimation (MNE) [29]. Although classical studies set **C** and **R** as identity matrices, here we used a modified version of MNE, in which **C** was computed from simulated MEG signals with a period of −100 to −1 ms, **R** was weighted by depth from the cortical surface, and the leadfield matrix was spatially whitened. We tested different *λ* values ranging from 10^−2^ to 10^3^ in exponential steps. We used MNE implemented in Brainstorm (http://neuroimage.usc.edu/brainstorm/).

#### L1-norm regularization

L2-norm regularization yields a cortical current broadly distributed over the cortical surface. Such a solution is often considered undesirable, because one of the purposes of MEG source estimation is to identify the brain areas related to the experimental conditions under investigation. As an MEG source estimation method that gives preference to more sparse cortical currents than MNE, we tested L1-norm regularization, also known as minimum current estimation (MCE; [30,31]) or least absolute shrinkage and selection operator (LASSO; [32]), whose objective function is written as

$$E\left(\hat{j}\right)=\frac{1}{2N}{\left|b-G\cdot \hat{j}\right|}_{2}^{2}+\lambda {\left|\hat{j}\right|}_{1}$$ (3)

where *λ* is a regularization parameter, and |**x**|_*p*_ represents the L*p*-norm of a vector **X**. L1-norm regularization promotes sparse solutions in which only small numbers of elements of $\hat{j}$ have non-zero values. In this formulation, *λ* should be smaller than $\lambda_{\max}=\frac{\max\left(\left|G^{T}\cdot b\right|\right)}{N}$ [33,34]; otherwise $\hat{j}$ becomes the zero vector. As *λ*_max_ was on the order of 10^−11^ in our simulation, we tested *λ* values smaller than the *λ*_max_, ranging from 10^−16^ to 10^−11^ in exponential steps. We used the L1-norm minimization implemented in the scikit-learn software package (http://scikit-learn.org/stable/).

#### Hierarchical Bayesian estimation

The above two methods add a constraint for the minimization of the L2 and L1-norm of $\hat{j}$, respectively. The constraint can be further improved if prior information on the locations of the MEG signal sources is available. Previous studies have shown that fMRI activity evoked by experimental conditions similar to those used to induce MEG signals can serve as prior information about the possible locations of MEG signal sources [35,36].

In this study, we used a hierarchical Bayesian model to introduce prior information on the MEG signal sources [36]. In this model, the prior information for $\hat{j}$ is introduced as a prior probability that follows a zero-mean Gaussian distribution with inverse variance **r**:

$$P\left(\hat{j}|r\right)\propto \exp\left(-\frac{1}{2}{\hat{j}}^{T}\cdot R\cdot \hat{j}\right)$$ (4)

where **R** = diag(**r**) and **r** is a ℝ^*M*×1^ vector whose elements $r_{i}^{-1}$ are hyperparameters denoting source variance. The inverse variance **r** is also treated as a random variable that follows a gamma distribution

$$P\left(r\right)=\prod_{i=1}^{M}\Gamma \left(r_{i}|{\bar{r}}_{0,i},\gamma_{0,i}\right)$$ (5)

where $\Gamma \left(r_{i}|{\bar{r}}_{0,i},\gamma_{0,i}\right)$ represents a gamma distribution with mean ${\bar{r}}_{0,i}$ and degrees of freedom *γ*_0,*i*_. The *γ*_0,*i*_ is also known as a confidence parameter. The prior distribution combined with this hyperprior distribution is equivalent to automatic relevance determination (ARD) prior [36–38]. Information about the fMRI activity is introduced to control ${\bar{r}}_{0,i}$, with a large source variance being more likely where larger fMRI activity is observed. The likelihood function is defined with the assumption that the MEG sensor noise follows a Gaussian distribution as

$$P\left(b|\hat{j}\right)\propto \exp\left(-\frac{1}{2\alpha }{\left\|b-G\cdot \hat{j}\right\|}^{2}\right)$$ (6)

where *α* indicates noise variance. Using Bayes’ theorem, the posterior probability distribution for the estimated cortical current can be described as

$$P\left(\hat{j},r|b\right)=\frac{P\left(b|\hat{j}\right)P\left(\hat{j}|r\right)P\left(r\right)}{P\left(b\right)}$$ (7)

where *P*(**b**) indicates the marginal likelihood defined as

$$P\left(b\right)=\int P\left(b|\hat{j}\right)P\left(\hat{j}\right)d\hat{j}.$$ (8)

Using $P\left(\hat{j},r|b\right)$, the posterior distribution of $\hat{j}$ can be obtained as

$$P\left(\hat{j}|b\right)=\int P\left(\hat{j},r|b\right)dr.$$ (9)

The cortical current is estimated by taking the expectation of the posterior distribution.

We conducted this hierarchical Bayesian estimation using variational Bayesian multimodal encephalography software (VBMEG; http://vbmeg.atr.jp/). For prior information, we set the magnitude of the fMRI activity to 1 for all vertices in V1 and IT and to 0 for all other vertices. Note that cortical current was estimated for all vertices on the whole cortical surface regardless of the prior information. The width of the time window for estimating source variance were set to 100 ms and the time window was shifted in 50-ms steps. We used a single value of the confidence parameter for all vertices (i.e., *γ*_0,*i*_ = *γ*_0_). Tested values were *γ*_0_ = 0 and that in the range from 10^−1^ to 10^3^ in exponential steps.

#### LCMV beamformer

The fourth representative method for MEG source estimation that we tested was the linearly constrained minimum variance beamformer (LCMV; [39]), which is based on a different concept from the regularization methods described above. LCMV constructs a spatial filter **w**_*i*_ for **b** to estimate the *i*th cortical current ${\hat{j}}_{i}$ under the constraint $w_{i}^{T}\cdot g_{i}=1$, where **g**_*i*_ is the column vector of the leadfield matrix corresponding to the *i*th vertex. Under this constraint, LCMV minimizes the power of ${\hat{j}}_{i}$. This minimization effectively suppresses the influence of spurious sources and sensor noise because the lower bound of the power of ${\hat{j}}_{i}$ should match the original cortical current as a result of the constraint. This procedure yields a spatial filter **w**_*i*_:

$$w_{i}=B^{-1}\cdot g_{i}\cdot {\left(g_{i}^{T}\cdot B^{-1}\cdot g_{i}\right)}^{-1}$$ (10)

where **B** indicates the covariance matrix of **b**. Because the signal-to-noise ratio of **b** can be low in an actual experiment, noise can significantly affect **w**_*i*_. Thus, **w**_*i*_ is normalized by noise variance as

$${\hat{w}}_{i}=\frac{w_{i}}{g_{i}^{T}\cdot C\cdot g_{i}}.$$ (11)

Thus, ${\hat{j}}_{i}$ can be obtained as

$${\hat{j}}_{i}={\hat{w}}_{i}^{T}\cdot b.$$ (12)

$\hat{j}$ can thus be estimated by calculating ${\hat{w}}_{i}$ for all vertices. In this study, **B** was computed using a 100-ms time window shifted with 50-ms steps. Noise covariance matrix **C** was computed from simulated MEG signals with a period of −100 to −1 ms. We performed the above procedure using custom written MATLAB (MathWorks, Natick, MA) programs.

In this study, MEG source estimation was conducted using all trials for each artificial experimental run independently to avoid using the same data twice in the training and test (double dipping) in leave-one-run-out cross-validation (see **Time-resolved decoding in each ROI** section).

### Evaluation of source estimation accuracy

To evaluate source estimation accuracy, we used the two indices of area under the precision-recall curve and correlation of spatial patterns. Note that because the value of the MEG sensor noise was set high to resemble actual measurements, these evaluations would have been strongly influenced by noise if based on the data from a single trial. We therefore averaged the estimated cortical current across all trials for each artificial experimental condition and used the averaged data for evaluations.

#### Area under the precision-recall curve

First, we used the area under the precision-recall curve (APR) to evaluate the accuracy of localization of source cortical current by the MEG source estimation methods. As we simulated the source cortical current as a distribution rather than a current dipole, it is suitable to evaluate the degree of overlap between the source ROIs and the source positions defined by the estimated cortical current. Representative methods for such an evaluation are the area under the receiver operating characteristic curve (AUC) [40] and APR [41,42]. A previous study showed that AUC is less sensitive than APR when the number of positive samples is significantly different from the number of negative ones [43]. As the numbers of vertices within (positive samples) and outside (negative samples) a source ROI differed substantially (120 vs. 14,882) in our simulation, we used APR instead of AUC. The detailed procedure to calculate APR is shown in Appendix A. The chance level of APR was calculated as 0.008. APR was calculated at the time when the source cortical current was at its maximum for each ROI (50 ms and 225 ms for V1 and IT, respectively) in each artificial experimental condition.

#### Correlation of spatial patterns

Second, we calculated the correlation coefficients between the original and estimated cortical currents to evaluate how accurately spatial patterns were restored by MEG source estimation. Spatial correlations were computed between the original and estimated cortical current patterns over vertices within each source ROI for each artificial experimental condition at 50 ms and 225 ms for V1 and IT respectively. We assumed that the spatial patterns of the original cortical current were restored if a significant correlation (*P* < 0.05, uncorrected) was observed.

### Time-resolved decoding in each ROI

We also used pattern classification analysis to evaluate whether the MEG source estimation methods restored information represented by differences in the spatial patterns of the original cortical currents. Pattern classification was performed to predict the artificial experimental conditions from the estimated cortical current at each time interval in each ROI on a trial-by-trial basis. We used the linear support vector machine (SVM; [44]) implemented in libsvm (http://www.csie.ntu.edu.tw/~cjlin/libsvm/) as a pattern classifier or “decoder”. The decoder’s prediction accuracy was evaluated by time-resolved leave-one-run-out cross-validation analysis [16,45], in which the decoder was trained with four out of five artificial experimental runs and tested with the remaining one at a particular time interval of 5 ms (see below about preprocessing of the estimated cortical current). This procedure was repeated until all artificial experimental runs were tested once at each time interval.

The estimated cortical current was preprocessed before decoding as follows. First, the time course of estimated cortical current was downsampled at each 5-ms interval with an equally-weighted smoothing window of 10 ms. Then, the downsampled data underwent z-score normalization across trials at each time interval for each vertex. The parameters for normalization (i.e., mean and standard deviation) were calculated using only a training data set and were then applied to both the training and test data sets. A decoder received the preprocessed data corresponding to each ROI as an input feature (ℝ^120×1^ vector, for all ROIs). Custom software programs partially based on the Brain Decoder Toolbox (http://www.cns.atr.jp/dni/download/brain-decoder-toolbox/) were written to perform the analysis.

### Searchlight decoding

MEG source estimation cannot reconstruct the original cortical current with perfect accuracy, and spurious cortical current may be found beyond the source ROIs. If this spurious cortical current has systematic differences related to the artificial experimental conditions, a decoder can capture these differences and predict the artificial experimental conditions, even from irrelevant brain areas far from the source ROIs.

To test this possibility, we performed searchlight decoding [46] to examine whether and how far such systematic differences in estimated cortical current spread beyond the source ROIs. The estimated cortical current at each vertex and its 119 neighborhood vertices were used as an input feature (ℝ^120×1^ vector, the same dimensions as for the ROI-based decoding). Decoding was performed for all vertices using the same procedure as that employed for the ROI-based decoding, but the time points only of 50 ms and 225 ms were tested in this analysis.

To assess how far the systematic differences had spread, we analyzed the relationship between the distance from the source ROI to a particular vertex and the prediction accuracy at that vertex. Distance was calculated from the center of mass of the source ROI. This calculation was performed separately for each hemisphere.

### Statistical significance of decoding analysis

We defined the statistical significance level of these decoding analyses (ROI-based and searchlight) with reference to the prediction accuracy obtained from the whole cortical area at 0 ms using searchlight decoding for each participant. As the MEG signals at 0 ms consisted of noise, prediction accuracy at this time served as a null distribution. We used the 99th percentile of the null distribution as the statistical significance level. We also used binomial distribution and permutation tests to define the significance level and examined whether the significance of decoding accuracy depends on the definition of the significance level. The significance levels of the permutation tests were defined as 99th percentile of 200 random permutations at each time point.

### Real data analysis

We also applied pattern classification analysis to real data. In this analysis, we used VBMEG as a source estimation method because it showed the most accurate source localization and pattern reconstruction in the simulations (see Results).

#### Participants

Four male participated in this experiment voluntarily. Each participant gave written informed consent before participating in the experiment, which was approved by the institutional review board of The University of Electro-Communications and ATR Brain Activity Imaging Center.

#### Visual stimuli

Pairs of wedges with white and black checkerboard patterns were used as stimuli. The center angle of the wedges was 30°, and the wedges rotated clockwise in 30° steps (S11(A) Fig). The stimuli were projected onto a translucent screen, subtending 18° × 18° of visual angle, with a gray background. Each trial consisted of the fixation period and the stimulus presentation period. In the fixation period, a fixation point consisting of black and white concentric circles was presented for 1,500 ms at the center of the screen. The white part of the fixation point changed to red 700–1,200 ms before the stimulus presentation period began. Participants were instructed to suppress eye blinks while the color of the fixation point was red. After the fixation period, the stimulus presentation period began and each stimulus was presented for 300 ms and was followed by a 300 ms inter-stimulus interval. The red part of the fixation point changed back to white when the stimulus presentation period ended. One run consisted of 20 trials, and a total of 12 runs were conducted for each participant.

#### MEG acquisition and preprocessing

We acquired MEG signals during the experiment using the same MEG system used to estimate head position for the simulation. MEG signals were acquired at 1000 Hz and a band-pass filter with 0.05 to 200 Hz was applied online. Electrooculography (EOG) was simultaneously obtained to allow rejection of trials corrupted by eye blinks and eye movement. Each participant’s head position in the MEG system was acquired before each run. We applied time-shifted principal component analysis [47] and a low-pass filter with a 100 Hz cutoff frequency to the measured MEG signals to reduce environmental and high-frequency noise. MEG signals corresponding to each stimulus presentation were epoched from −100 to 400 ms (0 ms was defined as stimulus onset) to constitute a trial. Channels whose MEG signal amplitude exceeded the range between −1 and 1 pT in more than 5% of all trials were discarded, as they were considered to be contaminated by noise. Trials were discarded from the following analysis if the EOG fell outside the range between −1 and 1 mV, or at least one MEG channel fell outside the range between −1 and 1 pT, as they were considered to be contaminated by eye blinks, eye movement, or noise.

#### Source estimation

Forward model

We used a cortical structural image of each participant’s whole head acquired using the Trio and Prisma scanners at the ATR Brain Activity Imaging Center with the acquisition parameters mentioned in simulations. The cortical surface of each participant’s structural image was polygonized with 15,002 vertices using FreeSurfer and VBMEG software such that the number of vertices matched to that used in the simulations. Head positions were acquired in the MEG system for each run and they were coregistered to the cortical surface. A leadfield matrix was then computed for each participant and each run using the boundary element method.

Source estimation

As prior information, we set the magnitude of fMRI activity to 1 for all vertices in the primary visual cortex (V1) and the higher visual cortex (HVC), leaving it as 0 for all other vertices. We chose these two ROIs to investigate whether spurious cortical current produced by MEG source estimation has systematic differences related to the experimental condition (see the section below). HVC was defined as a combination of the lateral occipital complex (LOC), fusiform face gyrus (FFA), and parahippocampal place area (PPA). V1, LOC, FFA, and PPA were identified for each participant using standard experimental procedures used in previous studies [48–51]. The width of the time window for estimating source variance was set to 10 ms, and the time window was shifted in 5-ms steps. We used *γ*_0,*i*_ = 10^2^ for all vertices because it showed the most accurate correlation between the estimated and original cortical current in the simulation. Source estimation was conducted using VBMEG for each participant and each run.

#### Time-resolved decoding in each ROI

To investigate whether the spurious cortical current contained systematic differences related to the experimental conditions in the real data, we conducted time-resolved decoding in each ROI. Pattern classification was performed to predict the experimental conditions from the estimated cortical current at each time point in V1 and HVC. Three pairs of wedges presented in the upper right and the lower left areas were labeled as condition 1, and the other three pairs of wedges presented in the upper left and the lower right areas were labeled as condition 2 (S1B Fig). As the latency of neural activity in V1 is expected to be shorter than that in HVC [52], the experimental condition is expected to be decoded from the estimated cortical current in V1 earlier than it can be decoded from that in HVC. Thus, if significant prediction accuracy is obtained in HVC at the same latency as in V1, we can assume that the spurious cortical current estimated in HVC has information about the experimental condition. In this analysis, we used a SVM as a classifier and its prediction accuracy was evaluated by leave-one-run-out cross-validation for each time point. Before decoding, the mean amplitude of the estimated cortical current during the baseline period, in which the stimulus-evoked neural activity did not appear in V1 (−100 to 40 ms), was subtracted from each trial and each vertex. The end of the baseline period was determined according to previous studies [52,53]. Z-score normalization was also applied, as in the simulation. As in the simulation, we defined the statistical significance level as the 99th percentile of prediction accuracy obtained from searchlight decoding at 0 ms (see **Searchlight decoding** section described below). We defined the onset latency of significant prediction accuracy in each ROI as the earliest time point from which the classifier showed significant prediction accuracy on at least 40 consecutive points (40 ms).

#### Time lag between changes in prediction accuracy and estimated cortical current

The time course of the estimated cortical current was also compared with that for the prediction accuracy of each ROI. To quantify the time course of the magnitude of the estimated cortical current for each ROI, we used F-statistics calculated by comparing the variance of the estimated cortical current across all vertices within the ROI at each time point with that during the baseline period. Before calculating the F-statistics, the estimated cortical current underwent subtraction of the mean amplitude of the baseline period followed by trial averaging for each vertex. Next, we calculated the cross-correlation between the time courses of the F-statistics and prediction accuracy. In this calculation, all the time points after the baseline period were used, and the lag in the maximum of the cross-correlation was acquired. Positive lag means that the time course of the prediction accuracy precedes that of the estimated cortical current.

### Searchlight decoding

We performed searchlight decoding to examine whether and how far systematic differences about experimental conditions spread in the estimated cortical current also for the real data. The estimated cortical current at each vertex and its 119 neighborhood vertices were used as an input feature (ℝ^120×1^ vector, the same dimensions as the simulation). Decoding was performed for all vertices using the same procedure as for the ROI-based decoding at the onset latency of prediction accuracy for time-resolved decoding in V1 for each participant because it is highly likely that V1 could be a major source that represents information corresponding to visual stimuli but few areas are active at that time. As in the simulation, we defined the significance level as the 99th percentile of prediction accuracy of searchlight decoding at 0 ms obtained from the whole cortical area for each participant. This significance level was also used for time-resolved decoding in each ROI.

## Results

### Simulation data analysis

#### Localization accuracy of source ROIs

**Fig 3:**
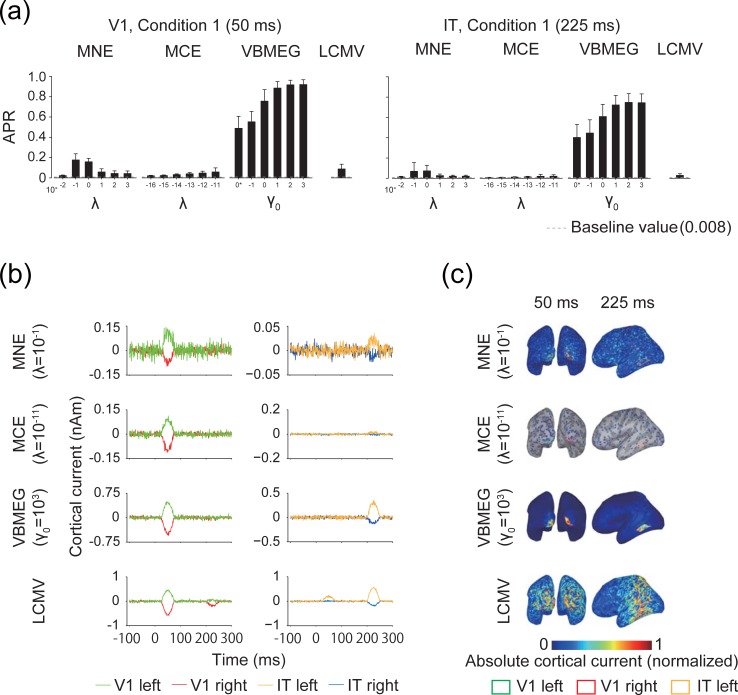
Accuracy of source localization. (a) Area under the precision-recall (APR) curves for V1 at 50 ms and IT at 225 ms in artificial experimental condition 1 (averaged across participants; error bars, s.d.; gray dashed lines, baseline value of APR). 0* indicates *γ*_0_ = 0. Results for artificial experimental condition 2 are shown in S2 Fig. (b) Time course of the mean estimated cortical current within V1 and IT with the hyperparameters that achieved the highest APR. The results for artificial experimental condition 1 for a single participant are shown as examples. (c) Absolute values of estimated cortical current with amplitude normalized to 0–1 for visibility. Vertices showing zero amplitude are set as transparent, and sulci (dark gray) and gyri (light gray) are visible on the maps. Results are shown for the same hyperparameters and participant as in (b).

First, we evaluated how accurately the MEG source estimation methods localized source cortical current using APR (Fig 3A; the results for V1 at 50 ms and IT at 225 ms in condition 1 are shown. The results of condition 2 are shown in S2 Fig). MNE, MCE, and VBMEG had at least one hyperparameter whose mean APR exceeded its baseline value in both ROIs. The mean APR of LCMV also exceeded its baseline value in both ROIs. VBMEG showed the highest APR, whereas MCE showed the lowest. We further illustrated the time course of the estimated cortical current within source ROIs for a single participant (Fig 3B) and the estimated cortical current maps by rendering them on the cortical surface of the same participant (Fig 3C). The time course of the estimated cortical current showed that the major sources were successfully estimated in the ROIs at the correct times, although they were missed in some cases (e.g., sources in IT at 200–250 ms by MCE) and small amplitudes were also found at the incorrect times (e.g., V1 at 200–250 ms and IT at 25–75 ms for LCMV). According to the results of the estimated cortical current maps, MNE, MCE, and VBMEG at 50 ms typically showed larger localized amplitudes around V1 than in other areas, whereas LCMV tended to produce a broad estimation (Fig 3C). At 225 ms, VBMEG showed better localization of a large amplitude around IT than other methods (Fig 3C). These results demonstrate that MEG source estimation can localize the major cortical current in the correct ROIs at the correct times, although some exceptions were found.

#### Spatial correlation between original and estimated cortical currents

**Fig 4:**
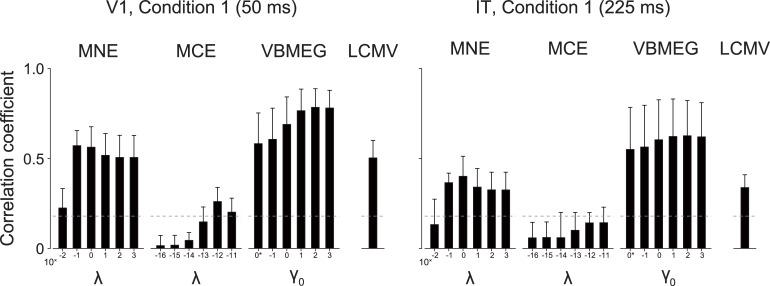
Correlation coefficient between patterns of original and estimated cortical current. Results for V1 at 50 ms and IT at 225 ms in artificial experimental condition 1 are shown (averaged across participants; error bars, s.d.; gray dashed lines, significance level [uncorrected *P* < 0.05]). 0* indicates *γ*_0_ = 0. Results for artificial experimental condition 2 are shown in S3 Fig.

We then evaluated the accuracy of restoration of the spatial patterns in the original cortical current. Correlation coefficients were calculated for spatial patterns between the original and estimated cortical currents within the source ROIs (Fig 4; only results for V1 and IT in condition 1 are shown, with the rest shown in S3 Fig). MNE showed the highest mean correlation at around *λ* = 10^−1^ to 10^0^ in both ROIs. MCE showed the highest correlation in V1 at *λ* = 10^−12^, but the mean correlations for IT did not exceed significance level. VBMEG generally showed higher correlations than the other source estimation methods and achieved the highest value around *γ*_0_ = 10^2^ in both ROIs. LCMV showed correlations comparable to those of MNE. These results indicate that all of the investigated MEG source estimation methods, except for MCE, can restore spatial patterns of the source cortical current that significantly correlate with the original ones.

#### Time-resolved decoding in each ROI

**Fig 5:**
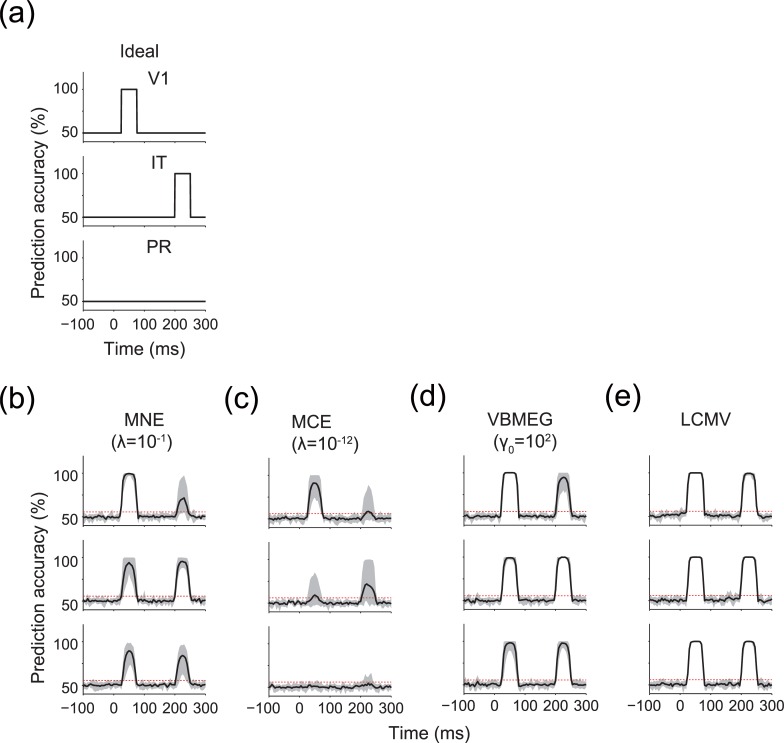
Time-resolved decoding in each ROI. (a) Ideal time courses of prediction accuracy. (b–e) Time courses of prediction accuracy for MNE, MCE, VBMEG, and LCMV, respectively. Results for the hyperparameters that achieved the highest correlation coefficient in V1 (see Fig 4) are shown. Solid lines indicate the mean prediction accuracy across participants. Shading indicates the 1st–99th percentiles of the prediction accuracy across participants. Red dashed lines indicate the mean significance level across participants. Results for other hyperparameters are shown in S4 Fig.

We further used pattern classification analysis to examine whether the MEG source estimation methods preserved the information represented by differences in the spatial patterns of the original cortical current. Because the difference was exclusively represented in V1 at 25–75 ms and in IT at 200–250 ms, only those particular combinations of ROI and time window were expected to show significant performance in prediction of the artificial experimental conditions (for ideal results, see Fig 5A).

The results showed that all MEG source estimation methods achieved significant prediction for these combinations of ROI and time window (in Fig 5B–5E, only results for the hyperparameter that achieved the highest correlation in V1 are shown [see Fig 4]; the full results are shown in S4 Fig). Thus, the represented information was preserved through the MEG source estimation.

However, significant prediction was also possible from the ROIs at non-informative time windows (i.e., V1 at 200–250 ms and IT at 25–75 ms), during which no differences were defined in the spatial patterns of the source cortical current, particularly for MNE, VBMEG, and LCMV. Furthermore, even in PR, where no source cortical current was provided at any time, significant prediction was possible for both time windows. MCE showed only a small increase in prediction accuracy for the non-informative combinations of ROI and time window, but it also achieved less accurate prediction than the other MEG source estimation methods with the informative combinations. We also performed the same analysis with addition of Gaussian noise *N* (0,1) to PR as source cortical current and examined whether such irrelevant activity influences decoding. The results showed that prediction accuracy was decreased but still remained above significance even in the case of non-informative combinations of ROI and time window (S5 Fig). These results demonstrate that information about artificial experimental conditions originally contained within a particular brain area can spatially spread to other brain areas through MEG source estimation. We call this phenomenon “information spreading.” Preliminary analyses also confirmed that these results were qualitatively similar if low-noise MEG signals or more complicated spatial patterns of source cortical current were used.

There was no qualitative difference between the significance levels calculated from the searchlight decoding at 0 ms (55.6±1.0%, presented in the figures), a binomial distribution (54.4%), and the permutation tests (56.1±0.8%, mean±s.d. across all time points in the trial). We also confirmed that no significant difference was observed in the number of significant time points across the three significance levels (*P* < 0.05, Kruskal-Wallis test) (S6 Fig). Because the statistical difference was not found to depend on the tested significance levels, we adopted the significance level calculated from the searchlight decoding at 0 ms in this paper.

#### Searchlight decoding and the spatial extent of information spreading

**Fig 6:**
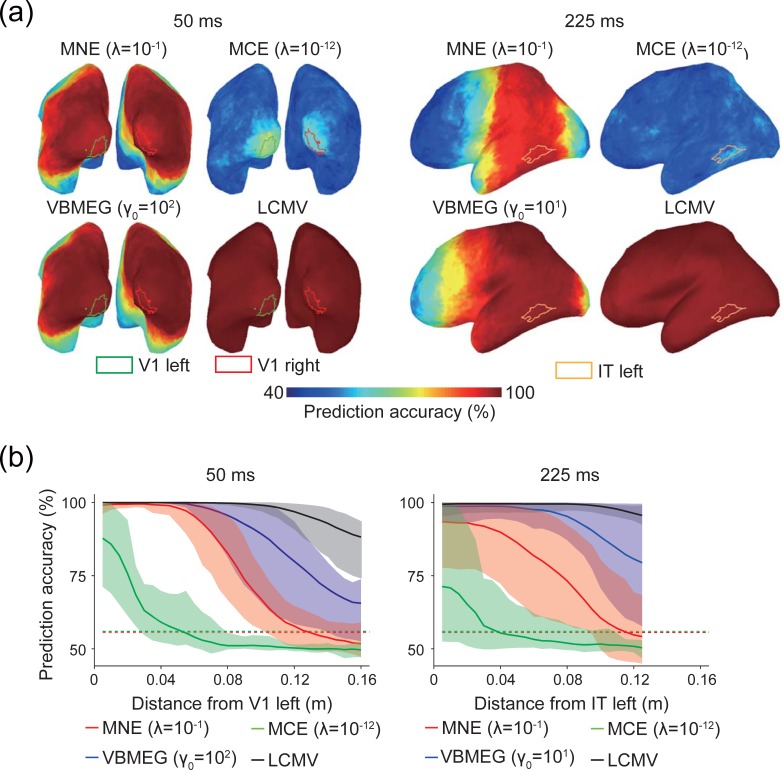
Maps of prediction accuracy obtained by searchlight decoding. Results for the hyperparameters that achieved the highest correlation coefficients (see Fig 4) are shown. (a) Maps of prediction accuracy at 50 ms and 225 ms. Results of a single participant are shown as examples. Colored lines delineate ROIs. (b) Spatial extent of information spreading at 50 ms (left) and 225 ms (right). The horizontal axes represent distance from the center of mass of V1 left (left) and IT left (right). Solid lines indicate mean prediction accuracy across participants. For an illustration purpose, prediction accuracy was averaged for each 5-mm distance bin. Shading indicates the 1st–99th percentiles of prediction accuracy across participants. Each dashed line indicates a significance level averaged across participants for each MEG source estimation method (they mostly overlap). Results for all hyperparameters in both hemispheres are shown in S7 Fig.

We conducted searchlight decoding to investigate the spatial extent of information spreading produced by MEG source estimation. Fig 6A shows maps of prediction accuracy for a single participant (the same participant shown in Fig 3B and 3C). Broad cortical areas showed near-perfect prediction accuracy for all MEG source estimation methods except MCE, whose prediction accuracy was much lower than that of the other methods, even within source ROIs (Fig 5). We quantified the spatial profile of prediction accuracy on the cortical surface (Fig 6B; results for the left hemisphere are shown. Full results are shown in S7 Fig). High prediction accuracy was observed in cortical areas distant from the source ROIs, particularly MNE, VBMEG, and LCMV, indicating that information spreading extended over wide cortical areas. MCE showed the narrowest information spreading, but its prediction accuracy was also the lowest. These results indicate that information spreading can potentially lead to false-positive identification of brain areas representing target information content, as significant decoding performance was found in irrelevant brain areas.

We further examined whether such wide information spreading was observed only in multivariate analysis or could also occur with univariate analysis. First, we calculated t-values for difference in amplitude of the estimated cortical current between artificial experimental conditions. Significant t-values were observed in cortical areas distant from the source ROIs (S8 Fig). Next, we conducted univariate searchlight decoding by using the mean value of estimated cortical current within each searchlight consisting of a target vertex and its 119 neighborhood vertices as an input feature. High prediction accuracy was observed in cortical areas distant from the source ROIs, but the prediction accuracy was lower than that of searchlight decoding using multivariate patterns as input features (S7 Fig and S9 Fig). For example, prediction accuracy for multivariate searchlight decoding stayed at 100% within at least 0.04 m from the source ROI for VBMEG irrespective of the hyperparameters whereas that for univariate searchlight decoding dropped rapidly as the distance from the source ROI. Other cases were also similar. These results indicate that information spreading can be observed if MEG source estimation is combined with univariate analysis (t-test and univariate decoding), but its effect is larger if combined with multivariate pattern analysis.

### Real data analysis

**Fig 7:**
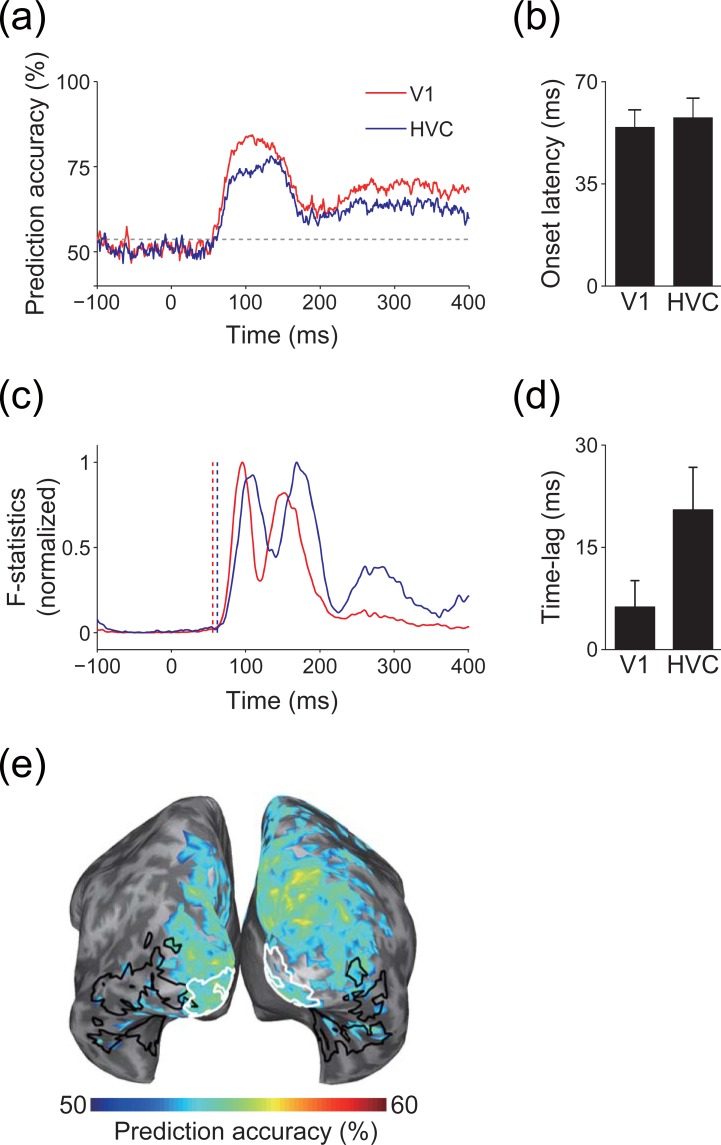
Information spreading in real data analysis. (a) Time course of prediction accuracy. Solid lines indicate prediction accuracy for V1 (red) and HVC (blue), respectively. Gray dashed line indicate significance level. Results of a single participant (participant 1) are shown as examples. (b) Mean onset latency of significant prediction accuracy for each ROI (error bars, s.d. across participants). (c) Time course of F-statistics. F-statistics are normalized to 0–1 for visibility. Vertical dashed lines indicate the onset of prediction accuracy for each ROI (red, V1; blue, HVC). Results of a single participant (participant 1) are shown as examples. (d) Mean lag of cross-correlation between the time course of prediction accuracy and F-statistics (error bars, s.d. across participants). Positive values indicate that prediction accuracy precedes the F-statistics. (e) Searchlight decoding. Cortical areas that showed significant prediction accuracy are colored. White and black enclosed areas are V1 and HVC, respectively. Results of a single participant (participant 1) are shown as an example. Results of other participants for (a), (c), and (e) are shown in S10A, S10B and S10C Fig, respectively.

To investigate whether information spreading also occurs in real data, we conducted pattern classification analysis of the estimated cortical current in each ROI to predict the visual stimuli presented to participants. Both ROIs showed a similar time course for prediction accuracy (Fig 7A), with almost the same onset latency for significant prediction accuracy between the ROIs (Fig 7B). These results are incompatible with electrophysiological knowledge suggesting that the latency of neural activity in HVC is longer than in V1 [52,53]. In contrast to the time course of prediction accuracy, the time course of the F-statistics in HVC was delayed from that in V1 (Fig 7C). Accordingly, the lag between prediction accuracy and F-statistics was smaller in V1 than in HVC, and prediction accuracy tended to precede F-statistics in HVC (Fig 7D). Thus, significant prediction accuracy was obtained before HVC was activated. In addition, although searchlight decoding was performed at a very short latency, at which V1 could be the truly informative ROI, significant prediction accuracy was obtained in other brain areas outside of V1 (Fig 7E). These results suggest that the significant prediction accuracy in HVC at this short latency was caused by spurious cortical current that might be originated from V1, indicating that information spreading also occurs in real data.

## Discussion

In this study, we evaluated whether MEG source estimation can restore information represented in original cortical current patterns. We found information spreading, which allowed decoders to achieve high prediction accuracy in irrelevant brain areas where no source was originally defined (Fig 5), even with accurate source localization (Figs 3 and 4). Searchlight decoding analysis further revealed that the effects of information spreading extended to widespread brain areas (Fig 6), especially for multivariate pattern analysis (S7 Fig and S9 Fig). In real data, significant prediction accuracy was obtained from V1 and HVC at the same latency, and other brain areas also showed significant prediction accuracy at a very short latency after the onset of the visual stimulation (Fig 7). These results indicate that MEG source estimation can restore information represented in patterns of original cortical current, but that this information spreads from informative brain areas to non-informative brain areas, demonstrating the possibility of false-positive interpretations when identifying brain areas that represent target information content based on the results of the decoding of estimated cortical current.

Previous studies have typically evaluated MEG source estimation methods in terms of the localization error and spatial dispersion of estimated cortical current (e.g. [2,4,5,7]). As the main focus of their source estimation was to minimize localization error and spatial dispersion, they did not pay much attention to the patterns of estimated cortical current within particular brain areas. This study has extended the evaluation of MEG source estimation methods and is the first to evaluate patterns of estimated cortical current. Our analyses suggest that the representative MEG source estimation methods tested here are imperfect with respect to their accuracy of localization of represented information content, even if they appear to provide highly accurate estimations of major activity in expected brain areas.

The origin of such imperfect localization of represented information (i.e., the cause for information spreading) probably lies in the underdetermined nature of MEG source estimation. MEG source estimation using a linear inverse method estimates the cortical current $\hat{j}$ from the observed magnetic field **b** using a linear inverse filter **L** as

$$\hat{j}=L\cdot b$$ (13)

and here, **b** can be rewritten using a noiseless linear observation model as

$$\hat{j}=L\cdot b=L\cdot G\cdot j=A\cdot j$$ (14)

where **A** (ℝ^*M*×*M*^ matrix) is called a resolution matrix [2]. **A** should be the identity matrix for perfect source reconstruction, but such reconstruction is impossible because the influence of different cortical sources on MEG channels can be very similar when the sources are located close to one another. Such a situation may be possible for all cortical locations, leading to non-zero off-diagonal elements of **A**. Accordingly, the original cortical current **j** is spatially spread by application of **A**, and spurious activity is estimated in irrelevant cortical areas. If such spurious activity shows a systematic difference between experimental conditions, even though its amplitude is very small, it can be informative enough for a pattern classifier to predict the corresponding experimental conditions because pattern classification analysis is more sensitive to slight differences in brain activity than conventional univariate analysis [20], resulting in information spreading (Figs 3B, 6 and 7).

In our simulation, we tested representative MEG source estimation methods based on L2- and L1-norm minimization to suppress such spurious cortical current. Both methods effectively diminished spurious cortical current in terms of its amplitude (Fig 3) but were insufficient to suppress information spreading (Fig 6). We also tested a model based on a hierarchical Bayesian framework using ARD prior. Even though its source localization accuracy was the highest among the methods tested (Fig 3A), the information spreading was qualitatively similar to that produced by the L2- and L1-norm minimization methods (Fig 6). Our results also indicate that the LCMV, another representative method for MEG source estimation, failed to diminish information spreading (Fig 6). Thus, none of the tested MEG source estimation methods provided a critical solution to suppress information spreading. Small but systematic differences in estimated cortical current patterns can yield confusing decoding performances.

Although we tested representative methods of MEG source estimation, other untested methods could possibly achieve accurate pattern estimation of original cortical current while suppressing information spreading. For example, a model combining L2- and L1-norm minimization, known as the elastic net [54], could work better than either model used individually because L1 and L2 often prefer solutions that are sparse and smooth, respectively (Fig 3B). A combination of these two methods could balance out these characteristics. VBMEG can also be updated by explicitly introducing similarity and group structures between parameters as their covariance. Similarity and group structure information could be inferred from actual neuroscientific knowledge about cortical parcellation [55] and structural/functional connectivity [8]. However, these updates increase the number of hyperparameters, and such model complications would present further difficulties in hyperparameter tuning.

As a potential method to reduce effects of information spreading, we attempted a signal leakage reduction based on orthogonalization, which has been proposed as a useful method to diminish artifactual zero-lag correlation in functional connectivity (Appendix B). The method reduced the correlation between estimated cortical current in two different ROIs but also decreased prediction accuracy even in an ROI containing informative source cortical current. Therefore, signal leakage reduction could be insufficient to identify truly informative ROIs while reducing information spreading.

However, this fact does not deny a possibility that signal leakage reduction based on another signal processing methods works efficiently to mitigate information spreading. In fact, a recent study showed that a method based on independent component analysis (ICA) can reduce signal leakage through MEG source estimation while preserving significant decoding performance to predict categories of presented visual stimuli [56]. Although the study evaluated effects of signal leakage reduction only in nearby ROIs, the results suggest that the ICA-based method might possess a potential to reduce signal leakage and information spreading over wide brain areas.

Although we tested only classification analysis in this study, it would be interesting to examine whether similar phenomena are observed for other MVPA methods. For example, representational similarity analysis (RSA; [57]) has been one of the most popular MVPA methods used in fMRI studies (e.g. [58,59]), and recent studies have successfully applied it to MEG signal analysis [60,61]. Those studies applied RSA to measured MEG signals in the sensor space, but it seems worthwhile to examine whether the same analysis can be performed for estimated cortical current because the relationship between brain areas and information representation can be discussed more straightforwardly. To this end, it is necessary to reveal whether the similarities between the original cortical current obtained in experimental conditions (i.e., the representational geometry) hold after application of source estimation. It is also important to clarify whether the representational geometry propagates between brain areas as information spreading. Preliminary analyses have been conducted in our recent study [62]. These analyses will further clarify the limitations and characteristics of MEG source estimation from a multivariate perspective, helping us to extract and closely investigate the information content represented in the spatiotemporal dynamics of human brain activity.

In summary, this study showed that spurious cortical current is inevitably generated through MEG source estimation and that even though its amplitude is negligibly small, it contains systematic differences related to experimental conditions. These differences can be easily detected by a pattern classifier, and the unexpected prediction accuracy may lead to false-positive interpretations of brain areas that represent information on the experimental conditions. Therefore, our results may assist to define the reliability of scientific findings based on MEG source estimation and multivariate pattern analysis.

## Appendix A. Calculation of APR

To calculate APR, we first converted $\hat{j}$ to an absolute normalized value ${\hat{j}}_{\mathrm{norm}}$ as

$${\hat{j}}_{\mathrm{norm},i}=\frac{\left|{\hat{j}}_{i}\right|}{\max\left(\left|\hat{j}\right|\right)}$$ (A1)

We shifted the threshold *β* in the interval [0 1], and if the *i*th vertex showed ${\hat{j}}_{\mathrm{norm},i}>\beta$, we judged the *i*th vertex as one of the source positions in ${\hat{j}}_{\mathrm{norm}}$. In this analysis, we defined a set of vertices of source ROIs as Θ (i.e., vertices of V1 or IT) and defined a set of other vertices as $\bar{\Theta }$ (i.e., vertices other than V1 or IT). The *i*th vertex was judged as

$$\cdot \;a\;\mathrm{true}\;\mathrm{positive}\;(\mathrm{TP})\;\mathrm{if}\;{\hat{j}}_{\mathrm{norm},i}\geq \beta \;\mathrm{and}\;i\in \Theta .$$

$$\cdot \;a\;\mathrm{false}\;\mathrm{positive}\;(\mathrm{FP})\;\mathrm{if}\;{\hat{j}}_{\mathrm{norm},i}\geq \beta \;\mathrm{and}\;i\in \bar{\Theta }.$$

We defined a set of TP vertices at *β* as TP(*β*) and the same for FP as FP(*β*). A precision–recall curve can be created by plotting recall (REC(*β*)) against precision (PREC(*β*)). REC(*β*) and PREC(*β*) were computed as

$$\mathrm{REC}\left(\beta \right)=\frac{\mathrm{size}\left(\mathrm{TP}\left(\beta \right)\right)}{\mathrm{size}\left(\Theta \right)},$$ (A2)

$$\mathrm{PREC}\left(\beta \right)=\frac{\mathrm{size}\left(\mathrm{TP}\left(\beta \right)\right)}{\mathrm{size}\left(\mathrm{TP}\left(\beta \right)\right)+\mathrm{size}\left(\mathrm{FP}\left(\beta \right)\right)}$$ (A3)

where size( ) indicates the number of elements in the set. The baseline value of APR [43] can be defined as

$$\mathrm{Baseline}\;\mathrm{value}=\frac{\mathrm{size}\left(\Theta \right)}{\mathrm{size}\left(\Theta \right)+\mathrm{size}\left(\bar{\Theta }\right)}.$$ (A4)

## Appendix B. Testing signal leakage reduction

When functional connectivity is analyzed using MEG signals, it is known that signal leakage of estimated cortical current by MEG source estimation causes zero-lag correlation between cortical areas. This is an unexpected artifact because neural signal conduction should yield non-zero time lag between functionally connected areas (except areas sharing common inputs). To solve this problem, several studies have proposed methods for signal leakage reduction [63–65]. Here, we investigated whether a similar method can also help to reduce information spreading.

The basic idea common to the signal leakage reduction methods [63–65] is orthogonalization of a time series of estimated cortical current in one source location relative to another. In the case of orthogonalization of ROIs containing multiple sources, principal component analysis (PCA) was applied to multiple time series of estimated cortical current in each ROI, and the orthogonalization was applied to one-dimensional time series constructed with coefficients of principal components corresponding to the majority of the variance of the estimated cortical current [65]. Thus, previous studies performed the orthogonalization in a one-to-one, univariate manner. However, such orthogonalization cannot be applied straightforwardly to our case because multivariate pattern analysis needs to account for distributed patterns of estimated cortical current.

To deal with this condition, we tested sequential orthogonalization, which was an extension of the previous orthogonalization method proposed in [63]. Suppose that we have ROI X and ROI Y, which consist of *M* and *N* vertices, respectively. Our objective is to orthogonalize the estimated cortical current in ROI Y relative to that in ROI X. Let **x**_*m*_ and **y**_*n*_ be time series of the *m*th vertex in ROI X and the *n*th vertex in ROI Y, respectively. **x**_*m*_ and **y**_*n*_ have the same length (ℝ^*T*×1^ vector, where *T* is length of the time series). The sequential orthogonalization was conducted as follows:

$$\begin{array}{l} \mathrm{Step}\;0:\;y_{n,0}=y_{n}\\ \mathrm{Step}\;1:\;y_{n,1}=y_{n,0}-x_{1}x_{1}{}^{+}y_{n,0}\\ \;y_{n,1}=y_{n,0}-x_{1}x_{1}{}^{+}y_{n,0}\\ \;\vdots \\ \mathrm{Step}\;m:\;y_{n,m}=y_{n,m-1}-x_{m}x_{m}{}^{+}y_{n,m-1}\\ \;\vdots \\ \mathrm{Step}\;M:\;y_{n,M}=y_{n,M-1}-x_{M}x_{M}{}^{+}y_{n,M-1} \end{array}$$ (B1)

where **x**_*m*_^+^ indicates the pseudo-inverse of **x**_*m*_.

If components parallel to each of **x**_1_…**x**_*M*_ are subtracted simultaneously as **y**_*n*,*M*_ = **y**_*n*_−**x**_1_**x**_1_^+^**y**_*n*_−**x**_2_**x**_2_^+^**y**_*n*_⋯–**x**_*M*_**x**_*M*_^+^**y**_*n*_, components correlated between **x**_1_…**x**_*M*_ are subtracted from **y**_*n*_ redundantly because **x**_1_…**x**_*M*_ are not orthogonal to each other. Sequential orthogonalization can circumvent this problem because, at step m, a component parallel to **x**_*m*−1_ has already been subtracted one step before, and thus, a component correlated between **x**_*m*_ and **x**_*m*−1_ cannot be subtracted anymore. Following this principle, a component parallel to each of **x**_1_…**x**_*M*_ is subtracted from **y**_*n*_ only once. By repeating this procedure for all vertices in ROI X, the signal components contained in ROI X will be subtracted from **y**_*n*_.

However, the orthogonalization method has ambiguity in its directionality. Because we cannot specify which cortical areas are truly or falsely informative before analysis, the directionality of orthogonalization cannot be determined. To deal with this situation, we can apply the orthogonalization bidirectionally (e.g., orthogonalize IT relative to V1 and vice versa) and compare which ROI better preserves information regarding the experimental conditions after orthogonalization.

We adopted this approach and first applied it to the simulation data. Specifically, we orthogonalized the estimated cortical current in IT relative to V1 and vice versa at 25–75 ms. The effects of orthogonalization were evaluated according to the correlation between the time series of estimated cortical current in V1 and the orthogonalized one in IT and vice versa. The estimated cortical current was averaged across trials for each artificial experimental condition, and correlations were calculated for all pairs of vertices between the two ROIs.

S11(A) Fig shows that the correlation between estimated cortical current in V1 and IT was significantly reduced after the orthogonalization for all tested MEG source estimation methods, irrespective of the directionality of the orthogonalization. These results suggest that orthogonalization of the time series of estimated cortical current removed contamination of the cortical current from other ROIs.

Then, we applied classification analysis to the orthogonalized estimated cortical current in V1 and IT at 50 ms, the middle of the corresponding time period. S11(B) Fig shows that prediction accuracy for both V1 and IT significantly dropped after the orthogonalization. These results suggest that orthogonalization can reduce the effects of information spreading but also ruins the information represented by patterns of estimated cortical current in truly informative ROIs.

Such information loss may come from inaccuracy of MEG source estimation. Because MEG source estimation is imperfect, estimated cortical current in IT may contain certain components that are correlated with source cortical current in both V1 and IT. Therefore, if V1 is orthogonalized relative to IT, estimated cortical current in V1 is recursively subtracted from itself. Such recursive subtraction may lead to the loss of informative components of source cortical current in V1, resulting in a significant drop in prediction accuracy.

To examine whether similar phenomena are observed in real data, we applied the same sequential orthogonalization procedure to the real data shown in Fig 7 and S10 Fig. We orthogonalized the estimated cortical current in HVC relative to that in V1 and vice versa. The orthogonalization was performed using a 50-ms time window, which was shifted with 50-ms steps to cover the whole time series.

The results were similar to those observed for the simulation. S11(C) Fig shows that the correlation between estimated cortical current in V1 and HVC significantly dropped after the orthogonalization. Prediction accuracy for the presented visual stimuli also significantly dropped to chance level at almost all times across both ROIs (S11(D) Fig). These results suggest that the method worked similarly: information loss caused by recursive subtraction also occurred in the real data. In addition, we confirmed that no significant difference was observed in the results presented in S11(D) Fig when the order of **x**_*m*_ in Eq (B1) was randomly shuffled 10 times, indicating that the order of orthogonalization does not matter.

In summary, these results imply that although the orthogonalization methods can reduce the similarity of estimated cortical current in two ROIs, it could be difficult to reduce the effects of information spreading while preserving the information contents represented in the truly informative ROIs.

## Supporting information

S1 Fig Visual stimuli used for the MEG experiment. (a) Pairs of wedges rotating clockwise in 30° steps. Black frames around the background are drawn for visibility (not shown in the actual experiment). (b) Correspondence between experimental conditions and labels. Three pairs of wedges presented in the upper right and lower left areas were labeled as condition 1, while the other three pairs of wedges presented in the upper left and lower right areas were labeled as condition 2.
(PDF)

S2 Fig Accuracy of source localization. APRs for V1 at 50 ms and IT at 225 ms in experimental condition 2 (averaged across participants; error bars, s.d.; gray dashed lines, baseline value of APR) are shown. 0* indicates *γ*_0_ = 0.
(PDF)

S3 Fig Correlation coefficient between patterns of original and estimated cortical current. Results for V1 at 50 ms and IT at 225 ms for artificial experimental condition 2 are shown (averaged across participants; error bars, s.d.; gray dashed lines, significance level [uncorrected *P* < 0.05]). 0* indicates *γ*_0_ = 0.
(PDF)

S4 Fig Time-resolved decoding in each ROI. (a–d) Time courses of the prediction accuracy for MNE, MCE, VBMEG, and LCMV, respectively. Solid lines indicate the mean prediction accuracy across participants. Shading indicates the 1st–99th percentiles of prediction accuracy across participants. Red dashed lines indicate the mean significance levels across participants.
(PDF)

S5 Fig Time-resolved decoding in each ROI with random noise source in PR. (a–d) Time courses of prediction accuracy for MNE, MCE, VBMEG, and LCMV, respectively. Solid lines indicate the mean prediction accuracy across participants. Shading indicates the 1st–99th percentiles of the prediction accuracy across participants. Results with random source in PR are shown in blue. Results corresponding to those shown in Fig 5B–5E are shown in black. Red dashed lines indicate the mean significance levels across participants.
(PDF)

S6 Fig Comparisons of difference in definition of statistical significance levels. The number of time points that showed significant prediction accuracy via time-resolved decoding in each ROI (averaged across participants; error bars, s.d.). Results for the hyperparameters that achieved (a) the highest and (b) second-highest correlation coefficients in V1 (see Fig 4) are shown. No significant difference was observed among the definition of statistical significance levels (*P* > 0.05, Kruskal-Wallis test).
(PDF)

S7 Fig Spatial extent of information spreading. (a, b) Results for V1 left and V1 right at 50 ms. (c, d) Results for IT left and IT right at 225 ms. The horizontal axes represent distance from the center of mass of each ROI. Solid lines indicate the mean prediction accuracy across participants. For an illustration purpose, prediction accuracy was averaged for each 5-mm distance bin. Shading indicates the 1st–99th percentiles of the prediction accuracy across participants. Each dashed line indicates the mean significance level across participants for each MEG source estimation method (they mostly overlap).
(PDF)

S8 Fig Spatial extent of t-value. (a, b) Results for V1 left and V1 right at 50 ms. (c, d) Results for IT left and IT right at 225 ms. The horizontal axes represent distance from the center of mass of each ROI. Solid lines indicate the mean t-value across participants. For an illustration purpose, t-value was averaged for each 5-mm distance bin. Shading indicates the 1st–99th percentiles of the t-value across participants. Each dashed line indicates a significance level (t-test, uncorrected *P* < 0.05; degrees of freedom, 499).
(PDF)

S9 Fig Spatial extent of information spreading (univariate). (a, b) Results for V1 left and V1 right at 50 ms. (c, d) Results for IT left and IT right at 225 ms. The horizontal axes represent distance from the center of mass of each ROI. Solid lines indicate the mean prediction accuracy across participants. For an illustration purpose, prediction accuracy was averaged for each 5-mm distance bin. Shading indicates the 1st–99th percentiles of the prediction accuracy across participants. Each dashed line indicates the mean significance level averaged across participants for each MEG source estimation method (they mostly overlap).
(PDF)

S10 Fig Information spreading in real data analysis. (a) Time courses of prediction accuracy for each participant. Solid lines indicate the prediction accuracy for V1 (red) and HVC (blue), respectively. Gray dashed lines indicate significance levels. (b) Time courses of F-statistics for each participant, normalized between 0 and 1 for visibility. Vertical dashed lines indicate the onset latency of significant prediction accuracy for each ROI (red, V1; blue, HVC). (c) Maps of prediction accuracy obtained by searchlight decoding for each participant. Brain areas that showed significant prediction accuracy were colored. White and black enclosed areas are V1 and HVC, respectively.
(PDF)

S11 Fig Test of signal leakage reduction. (a) Change of correlation by orthogonalization in simulation data (averaged across participants; error bars, s.d.). (b) Change of prediction accuracy by orthogonalization in simulation data (averaged across participants; error bars, s.d.;). (c) Change of correlation by orthogonalization in real data (averaged across participants; error bars, s.d.). Results of 51–100 ms and 101–150 ms are shown as representative examples. (d) Time courses of prediction accuracy after orthogonalization. Solid lines indicate the prediction accuracy for V1 (red) and HVC (blue) of each participant. Gray dashed lines indicate significance levels.
(PDF)

S1 File Individual data points from APR analysis (Fig 3 and S2 Fig). (XLSX)

S2 File Individual data points from correlation analysis (Fig 4 and S3 Fig). (XLSX)

S3 File Individual data points from time-resolved decoding in each ROI (Fig 5 and S4 Fig). (XLSX)

S4 File Individual data points from searchlight decoding (Fig 6 and S7 Fig). (XLSX)

S5 File Individual data points from real data analysis (Fig 7 and S9 Fig). (XLSX)
