# Supplementary figures and images for: Information spreading by a combination of MEG source estimation and multivariate pattern classification
Source: PLoS One. 2018 Jun 18;13(6):e0198806. doi: 10.1371/journal.pone.0198806 (PMC6005563; doi:10.1371/journal.pone.0198806)

(a)

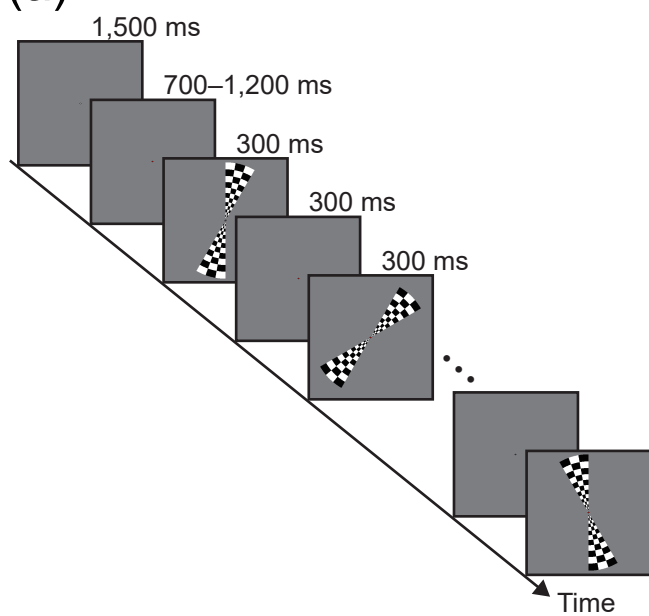

(b)

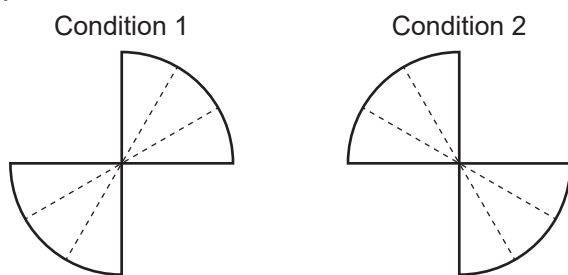

Supplement: S1 Fig — (a) Pairs of wedges rotating clockwise in 30° steps. Black frames around the background are drawn for visibility (not shown in the actual experiment). (b) Correspondence between experimental conditions and labels. Three pairs of wedges presented in the upper right and lower left areas were labeled as condition 1, while the other three pairs of wedges presented in the upper left and lower right areas were labeled as condition 2. (PDF) [file pone.0198806.s001.pdf]

V1, Condition 2 (50 ms)

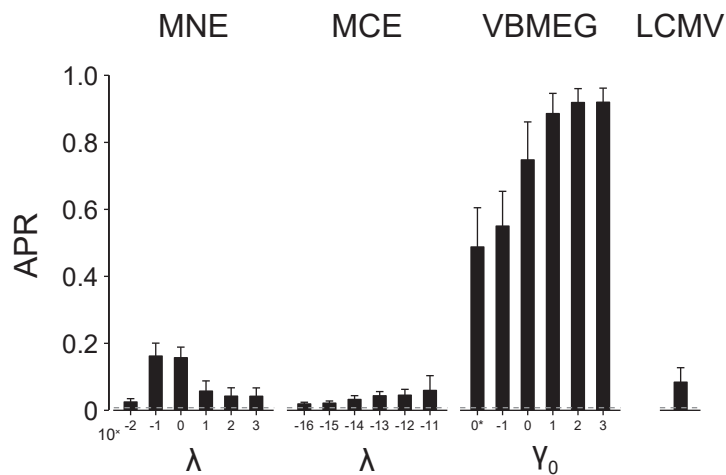

IT, Condition 2 (225 ms)

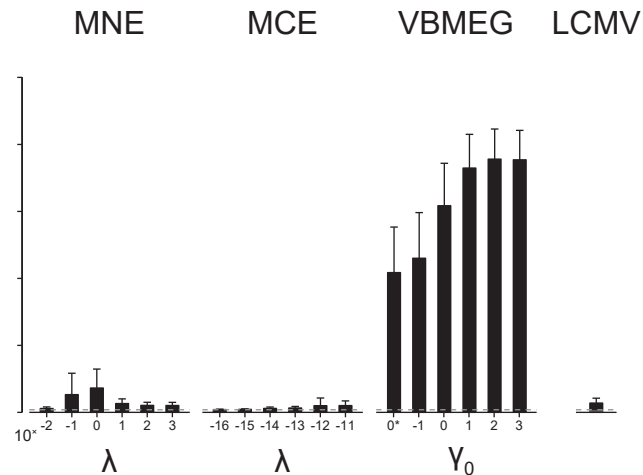

--- Baseline value (0.008)

Supplement: S2 Fig — APRs for V1 at 50 ms and IT at 225 ms in experimental condition 2 (averaged across participants; error bars, s.d.; gray dashed lines, baseline value of APR) are shown. 0* indicates γ0 = 0. (PDF) [file pone.0198806.s002.pdf]

V1, Condition 2 (50 ms)

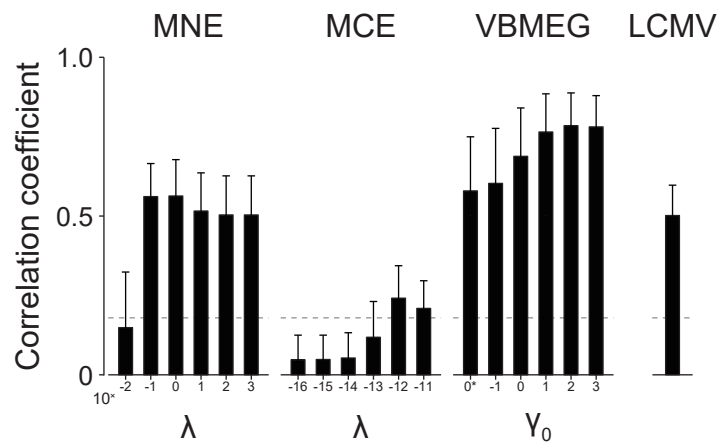

IT, Condition 2 (225 ms)

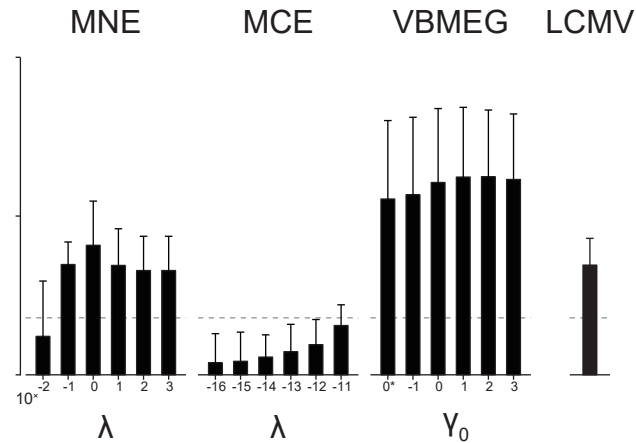

Supplement: S3 Fig — Results for V1 at 50 ms and IT at 225 ms for artificial experimental condition 2 are shown (averaged across participants; error bars, s.d.; gray dashed lines, significance level [uncorrected P < 0.05]). 0* indicates γ0 = 0. (PDF) [file pone.0198806.s003.pdf]

(a)

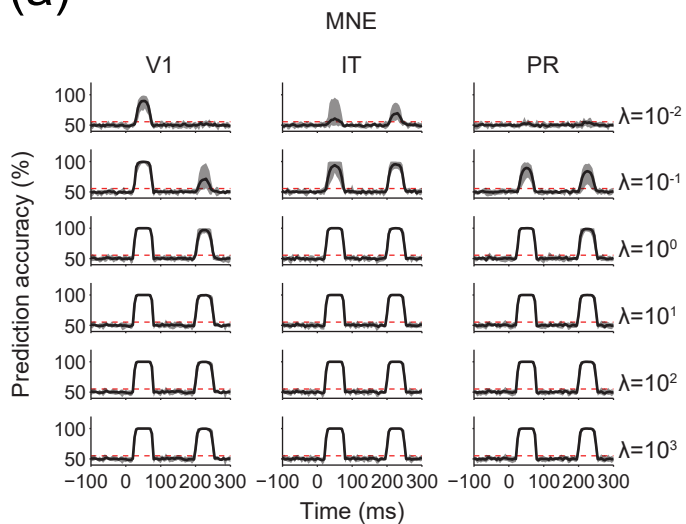

(b)

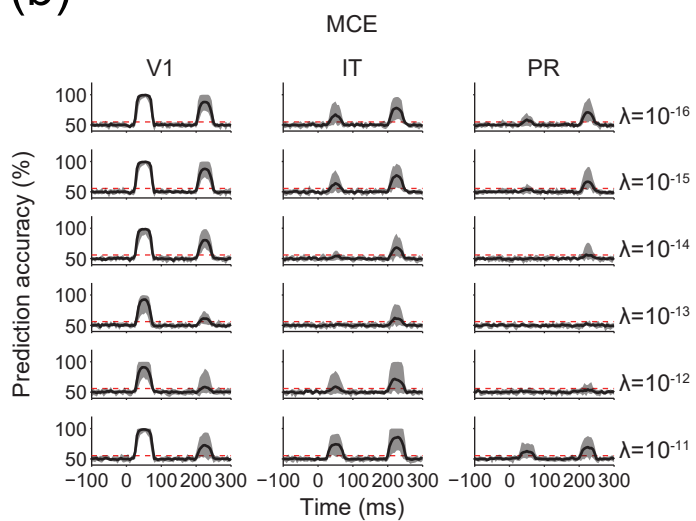

(c)

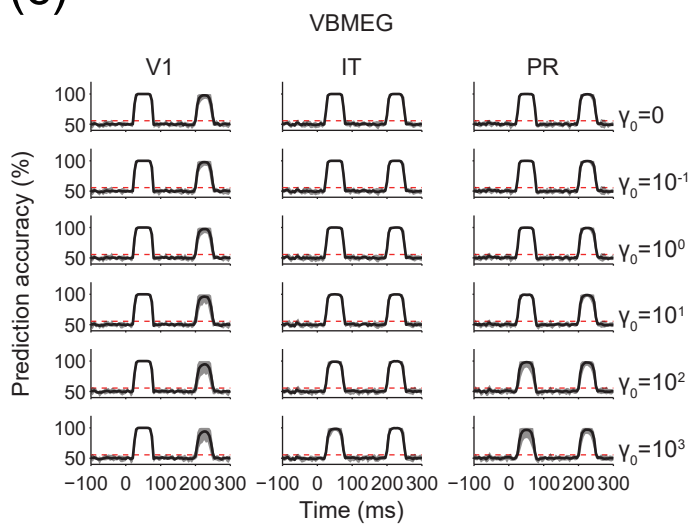

(d)

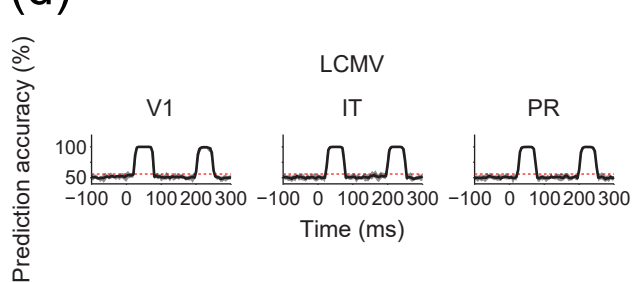

Supplement: S4 Fig — (a–d) Time courses of the prediction accuracy for MNE, MCE, VBMEG, and LCMV, respectively. Solid lines indicate the mean prediction accuracy across participants. Shading indicates the 1st–99th percentiles of prediction accuracy across participants. Red dashed lines indicate the mean significance levels across participants. (PDF) [file pone.0198806.s004.pdf]

(a)

MNE  
( $\lambda=10^{-1}$ )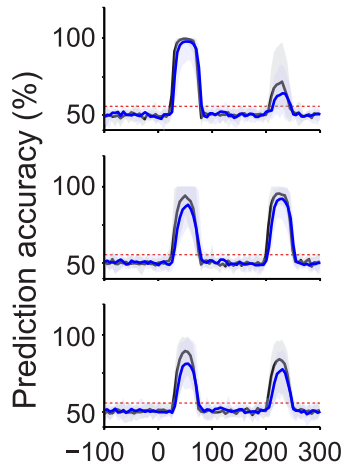

(b)

MCE  
( $\lambda=10^{-12}$ )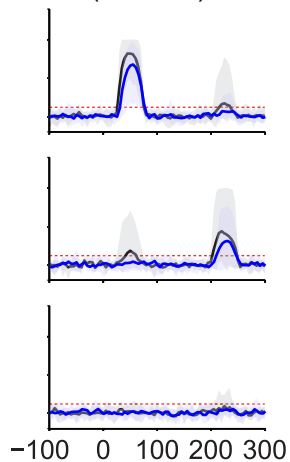

(c)

VBMEG  
( $\gamma_0=10^2$ )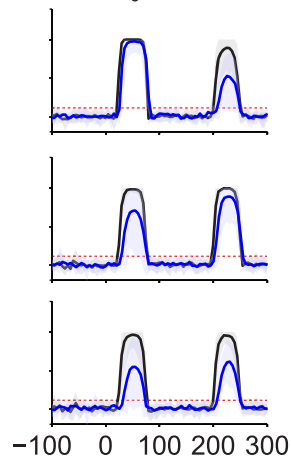

(d)

LCMV

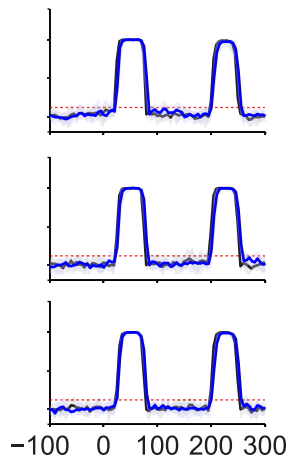

Time (ms)

Supplement: S5 Fig — (a–d) Time courses of prediction accuracy for MNE, MCE, VBMEG, and LCMV, respectively. Solid lines indicate the mean prediction accuracy across participants. Shading indicates the 1st–99th percentiles of the prediction accuracy across participants. Results with random source in PR are shown in blue. Results corresponding to those shown in Fig 5B–5E are shown in black. Red dashed lines indicate the mean significance levels across participants. (PDF) [file pone.0198806.s005.pdf]

(a)

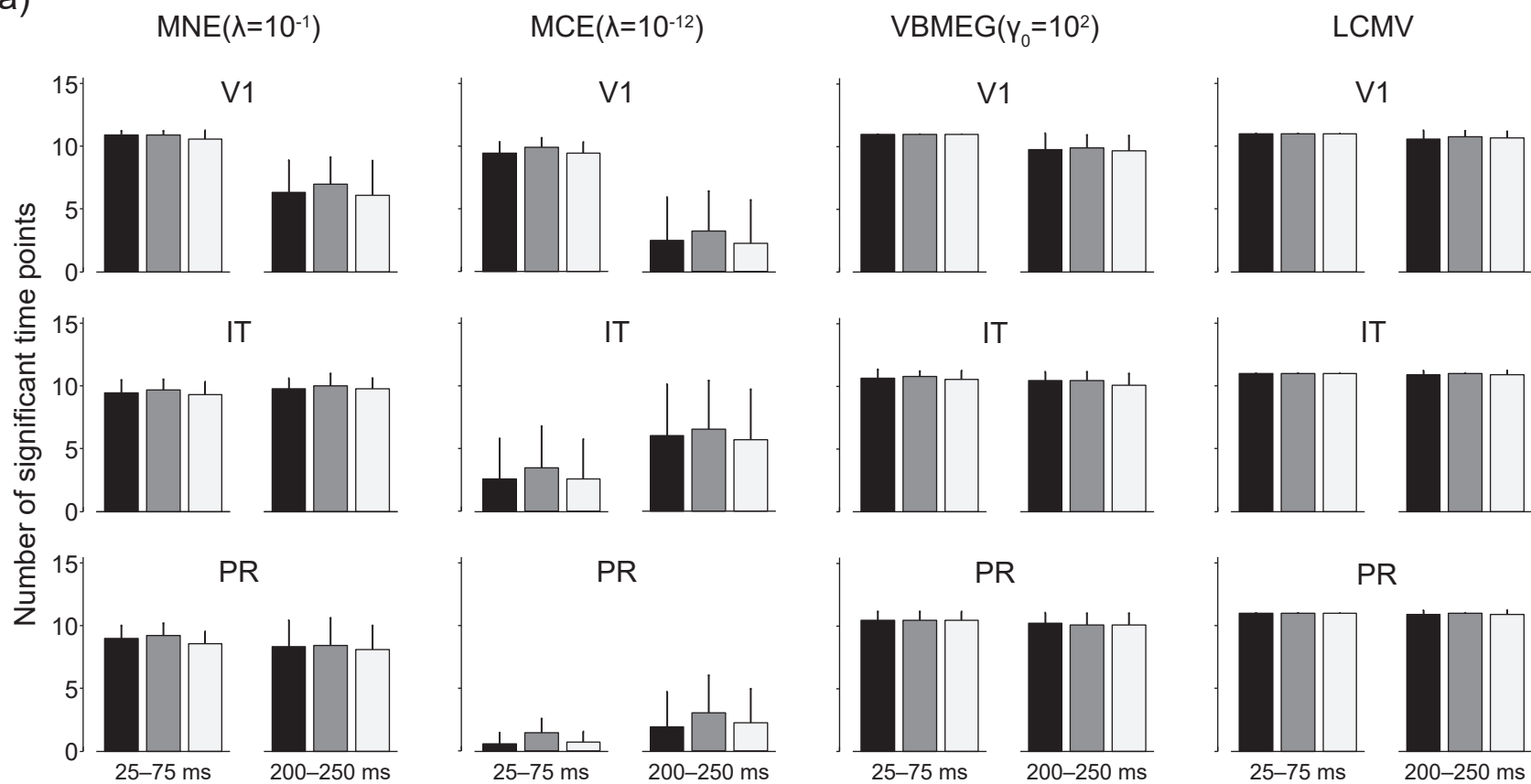

(b)

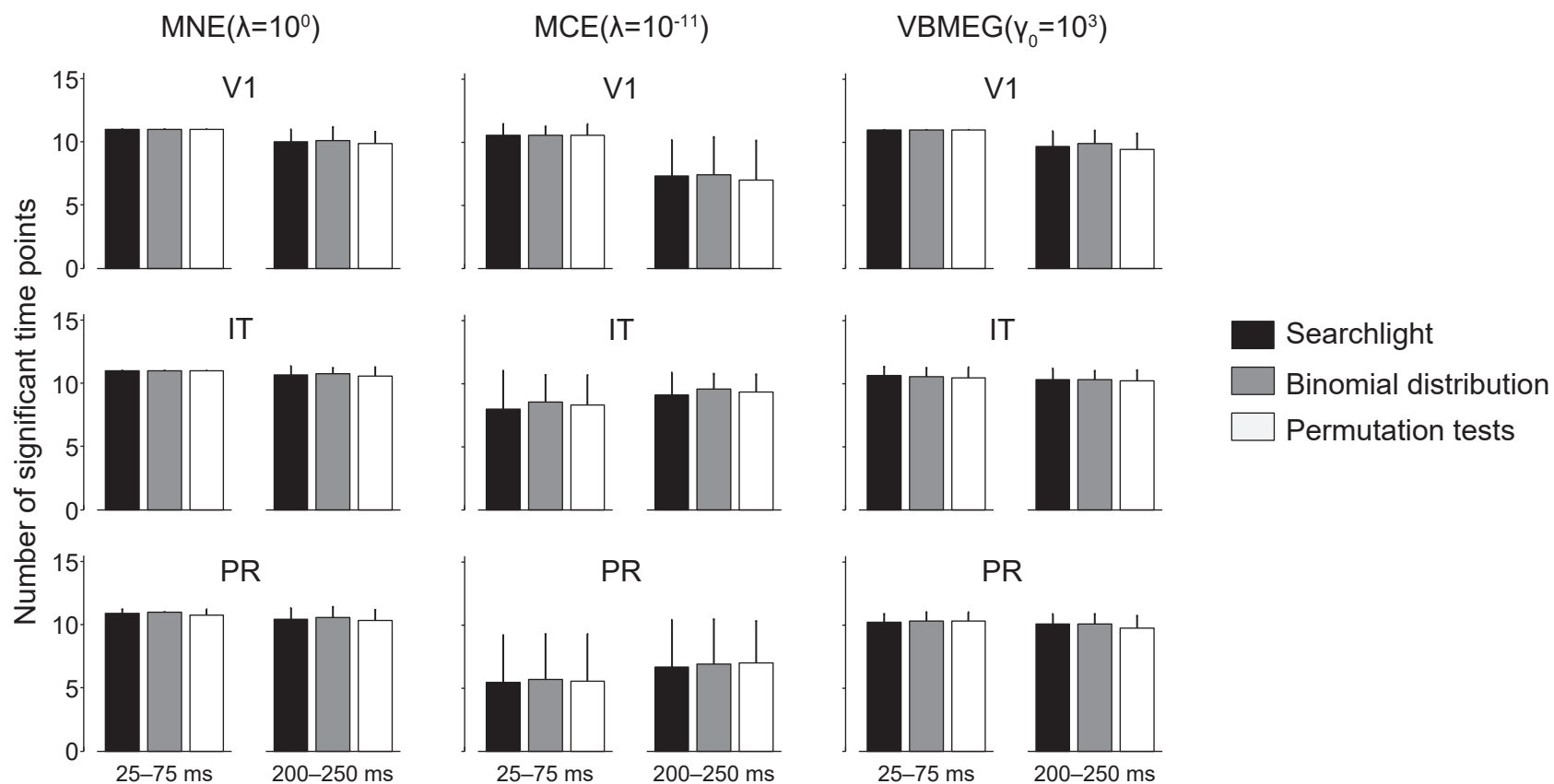

Supplement: S6 Fig — The number of time points that showed significant prediction accuracy via time-resolved decoding in each ROI (averaged across participants; error bars, s.d.). Results for the hyperparameters that achieved (a) the highest and (b) second-highest correlation coefficients in V1 (see Fig 4) are shown. No significant difference was observed among the definition of statistical significance levels (P > 0.05, Kruskal-Wallis test). (PDF) [file pone.0198806.s006.pdf]

(a)

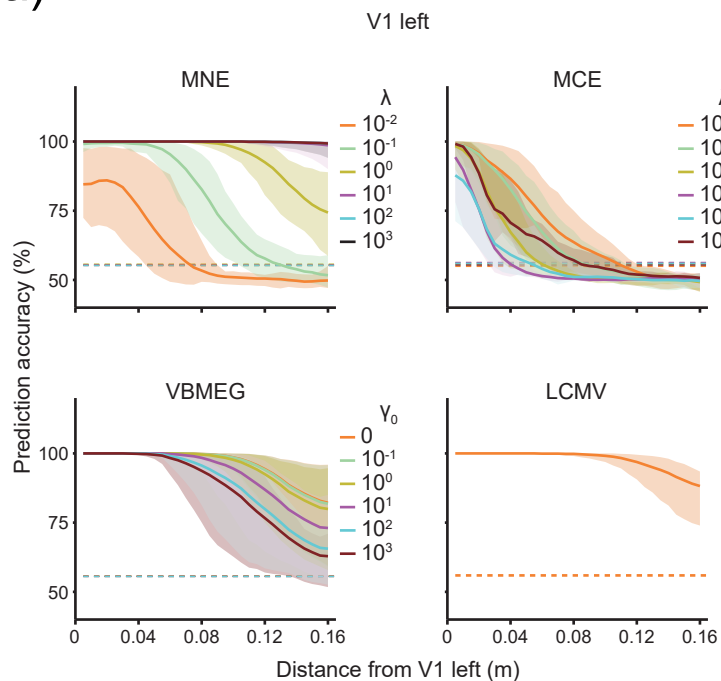

(b)

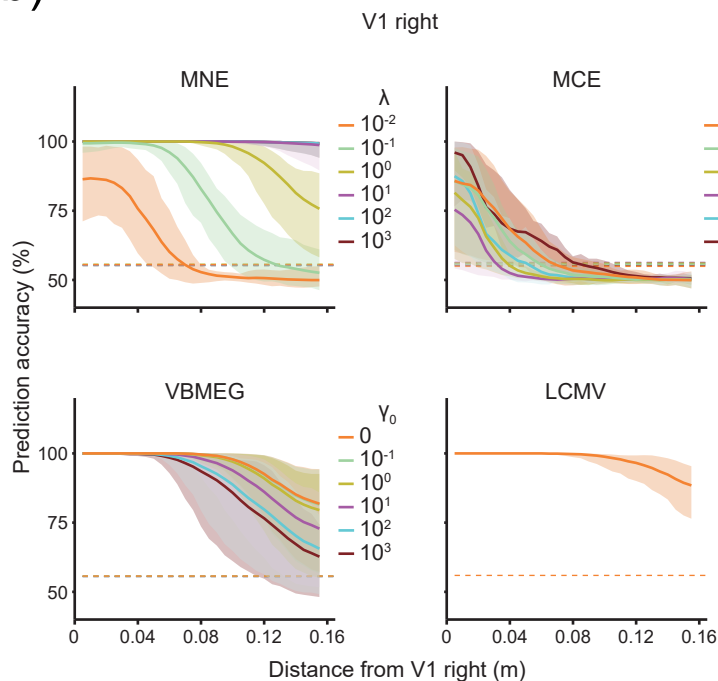

(c)

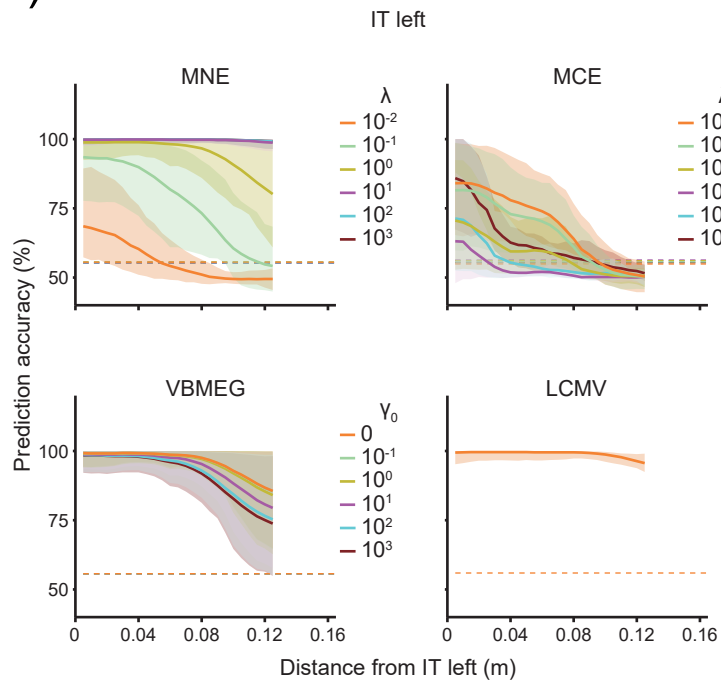

(d)

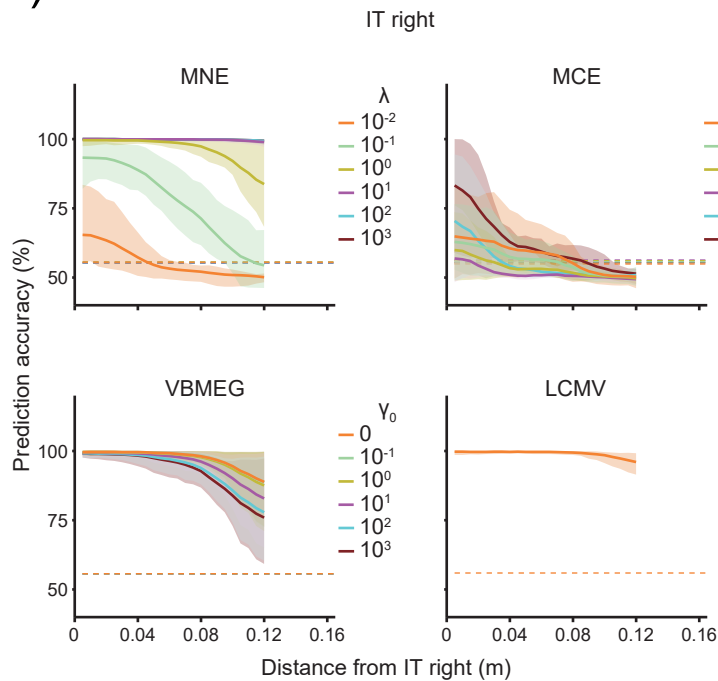

Supplement: S7 Fig — (a, b) Results for V1 left and V1 right at 50 ms. (c, d) Results for IT left and IT right at 225 ms. The horizontal axes represent distance from the center of mass of each ROI. Solid lines indicate the mean prediction accuracy across participants. For an illustration purpose, prediction accuracy was averaged for each 5-mm distance bin. Shading indicates the 1st–99th percentiles of the prediction accuracy across participants. Each dashed line indicates the mean significance level across participants for each MEG source estimation method (they mostly overlap). (PDF) [file pone.0198806.s007.pdf]

(a)

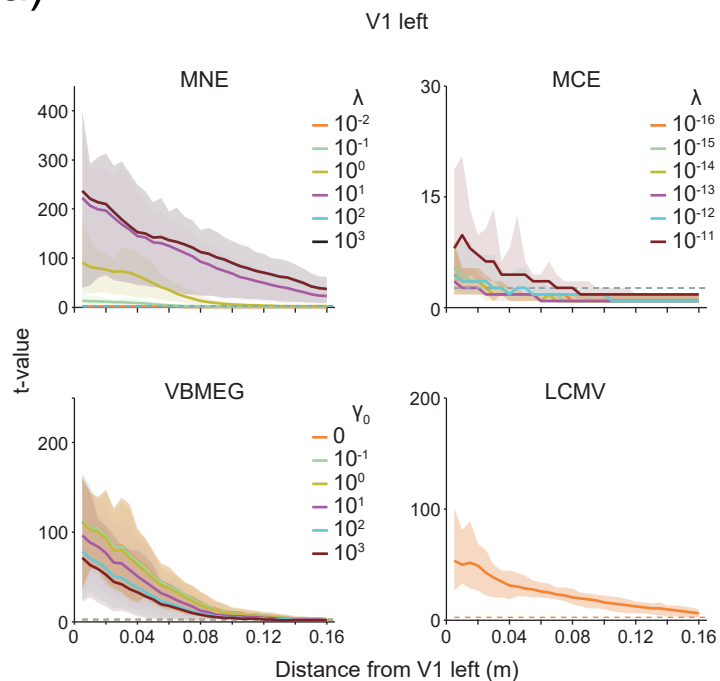

(b)

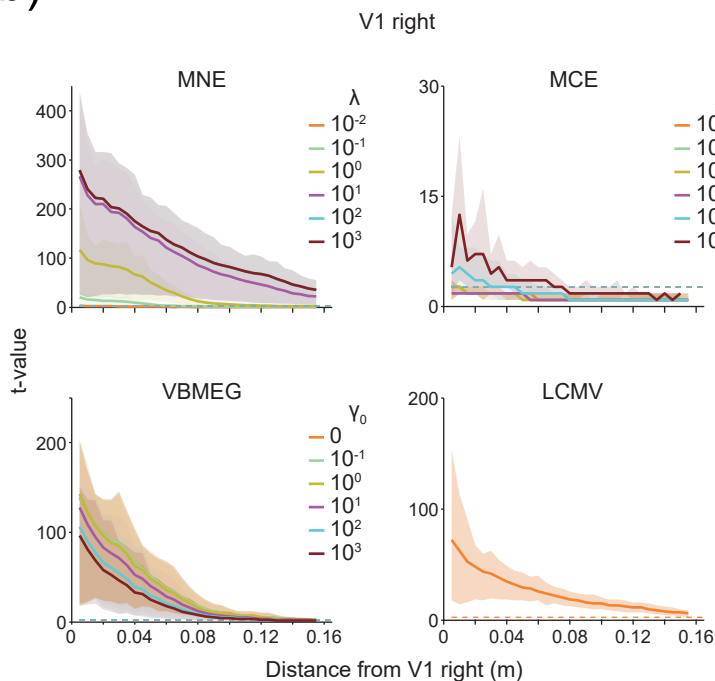

(c)

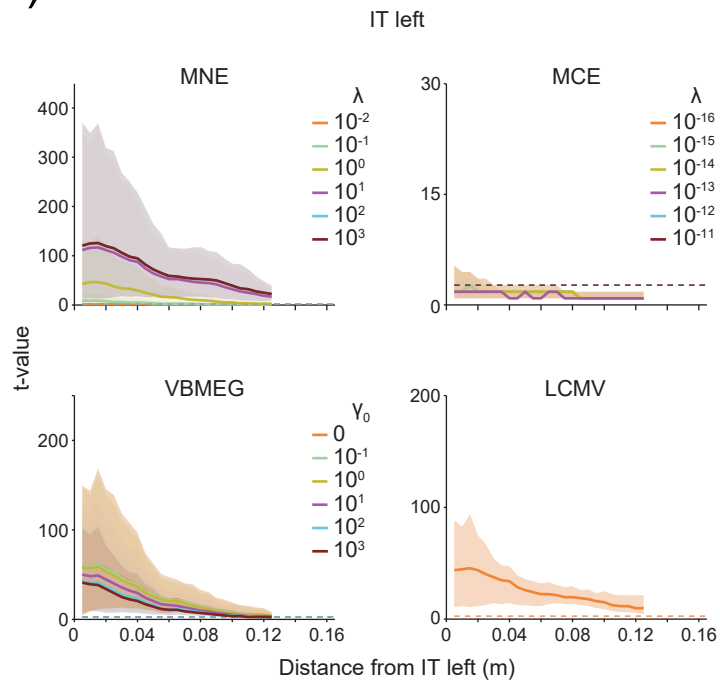

(d)

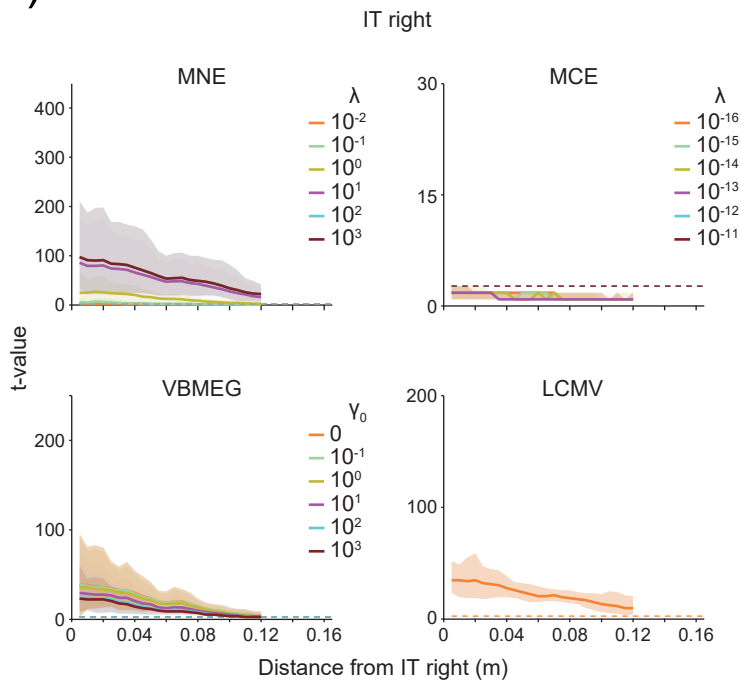

Supplement: S8 Fig — (a, b) Results for V1 left and V1 right at 50 ms. (c, d) Results for IT left and IT right at 225 ms. The horizontal axes represent distance from the center of mass of each ROI. Solid lines indicate the mean t-value across participants. For an illustration purpose, t-value was averaged for each 5-mm distance bin. Shading indicates the 1st–99th percentiles of the t-value across participants. Each dashed line indicates a significance level (t-test, uncorrected P < 0.05; degrees of freedom, 499). (PDF) [file pone.0198806.s008.pdf]

(a)

V1 left

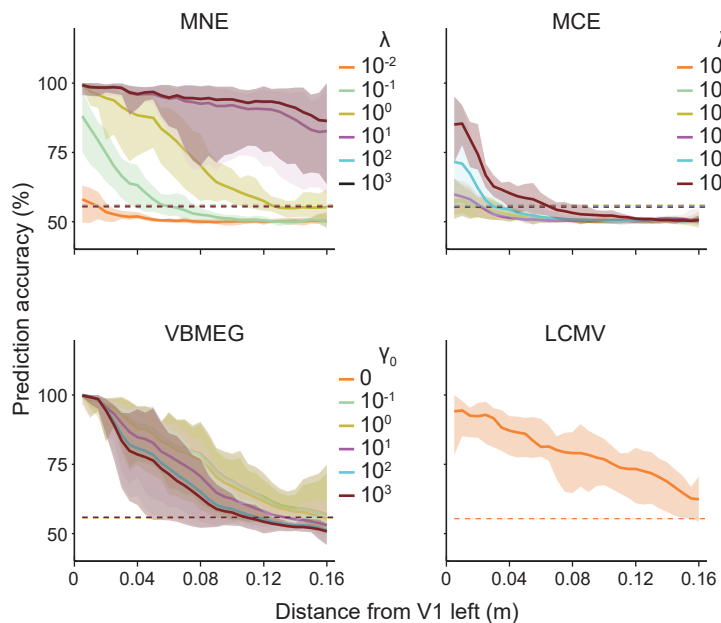

(b)

V1 right

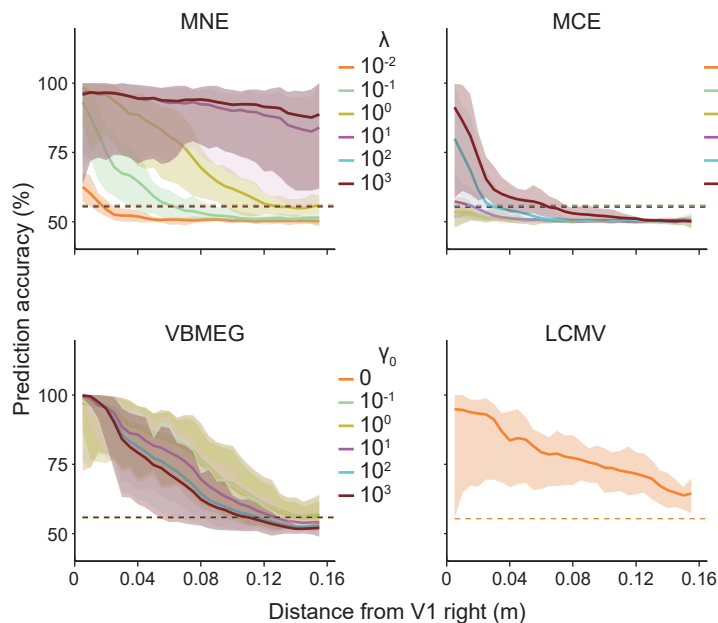

(c)

IT left

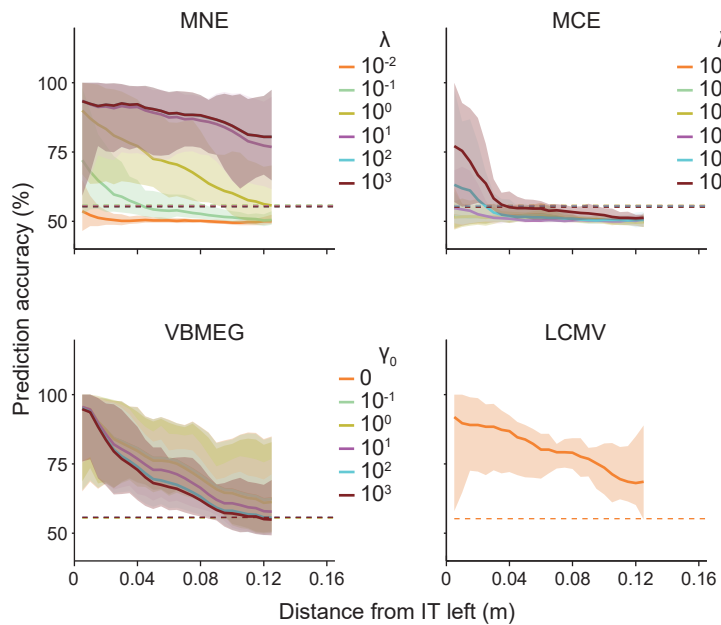

(d)

IT right

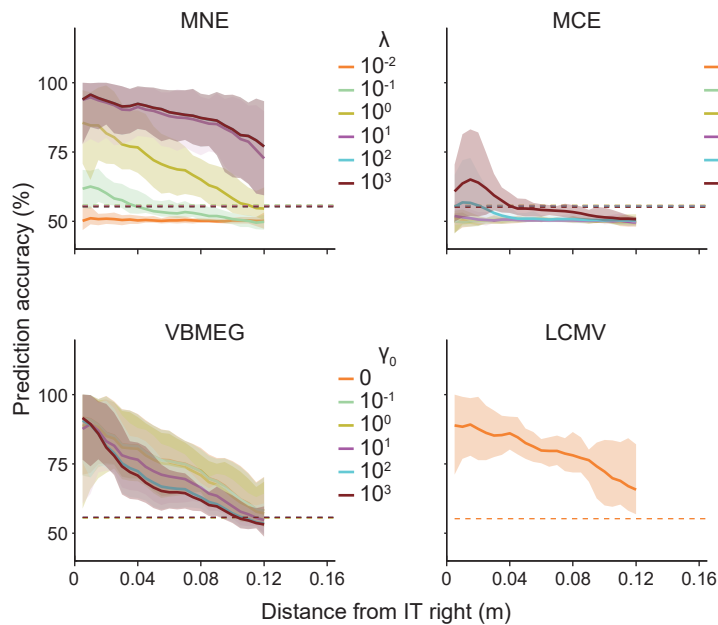

Supplement: S9 Fig — (a, b) Results for V1 left and V1 right at 50 ms. (c, d) Results for IT left and IT right at 225 ms. The horizontal axes represent distance from the center of mass of each ROI. Solid lines indicate the mean prediction accuracy across participants. For an illustration purpose, prediction accuracy was averaged for each 5-mm distance bin. Shading indicates the 1st–99th percentiles of the prediction accuracy across participants. Each dashed line indicates the mean significance level averaged across participants for each MEG source estimation method (they mostly overlap). (PDF) [file pone.0198806.s009.pdf]

(a)

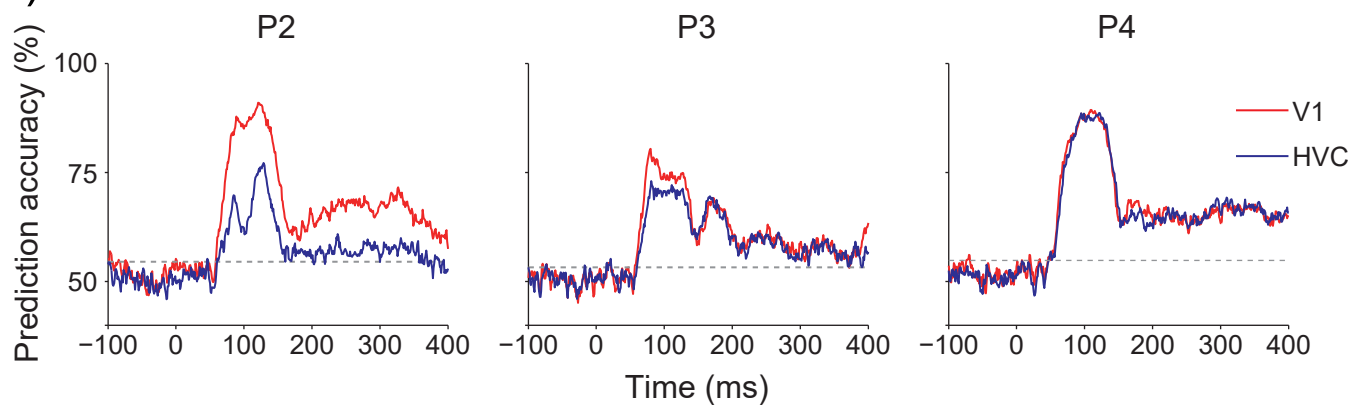

(b)

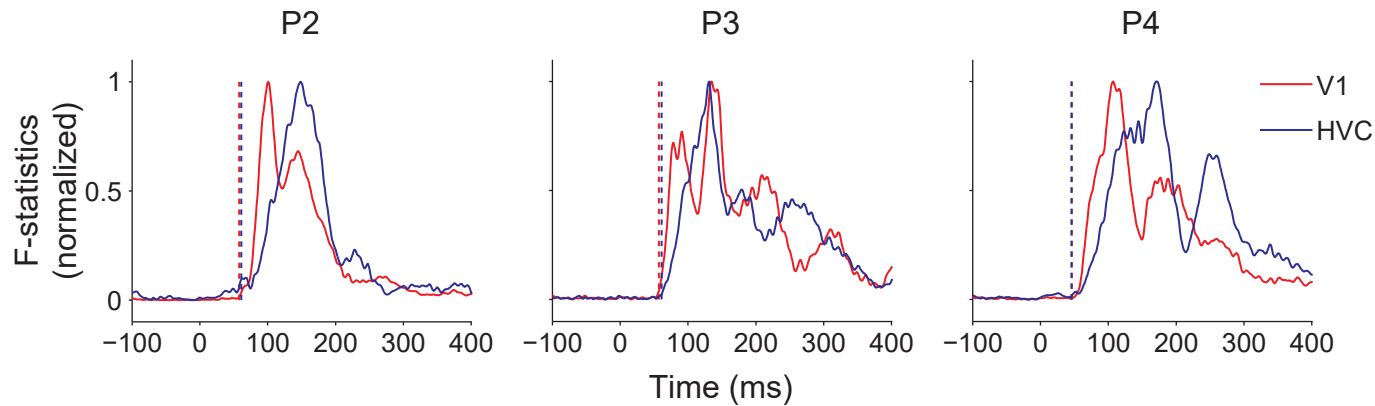

(c)

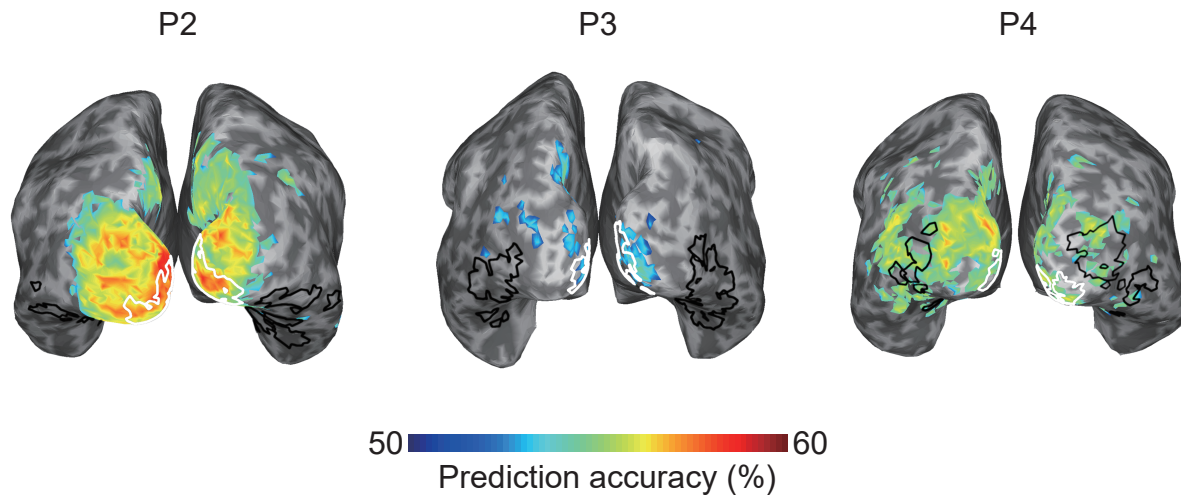

Supplement: S10 Fig — (a) Time courses of prediction accuracy for each participant. Solid lines indicate the prediction accuracy for V1 (red) and HVC (blue), respectively. Gray dashed lines indicate significance levels. (b) Time courses of F-statistics for each participant, normalized between 0 and 1 for visibility. Vertical dashed lines indicate the onset latency of significant prediction accuracy for each ROI (red, V1; blue, HVC). (c) Maps of prediction accuracy obtained by searchlight decoding for each participant. Brain areas that showed significant prediction accuracy were colored. White and black enclosed areas are V1 and HVC, respectively. (PDF) [file pone.0198806.s010.pdf]

(a)

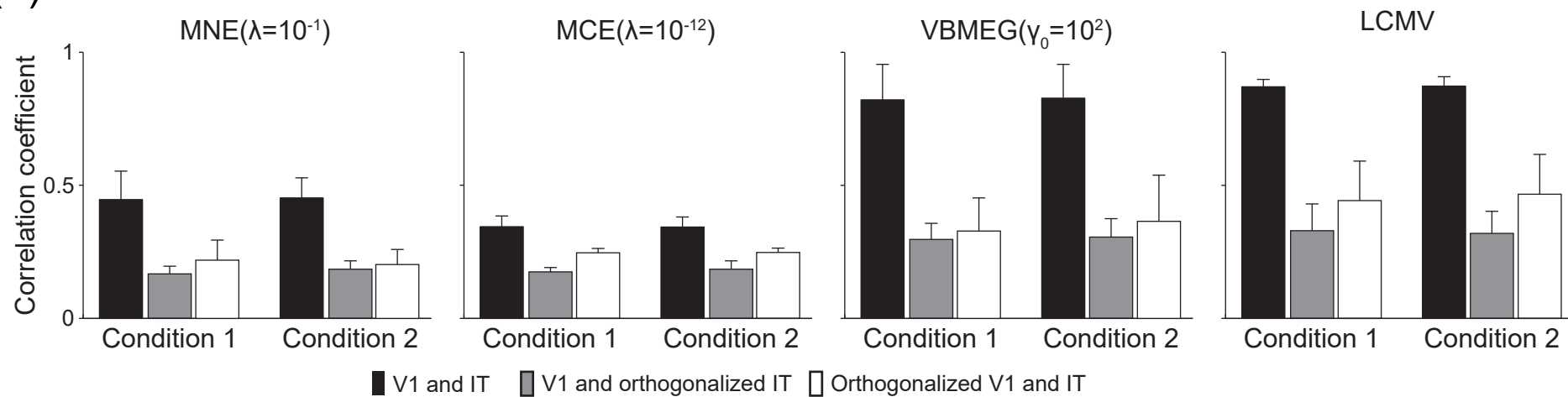

(b)

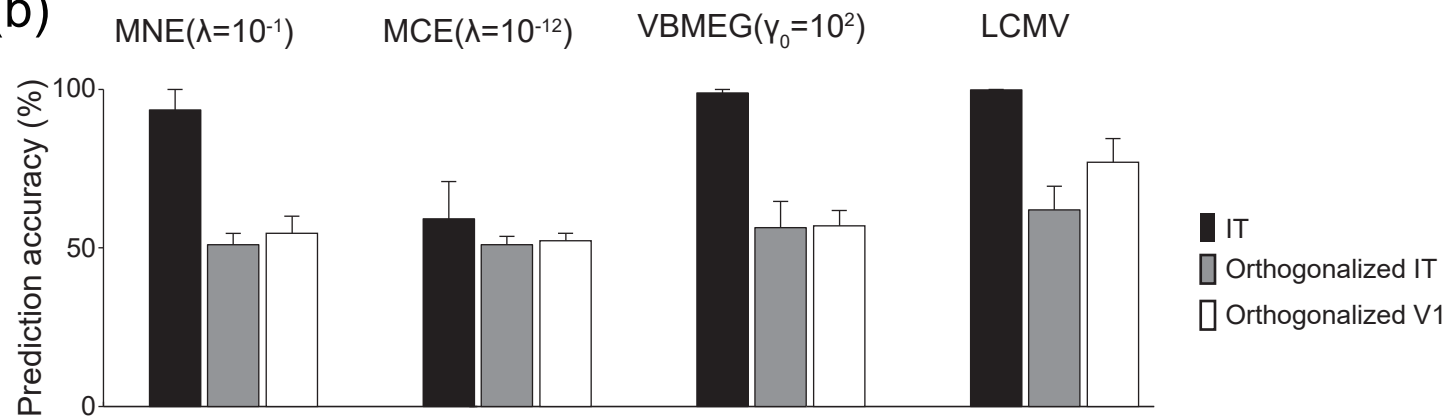

(c)

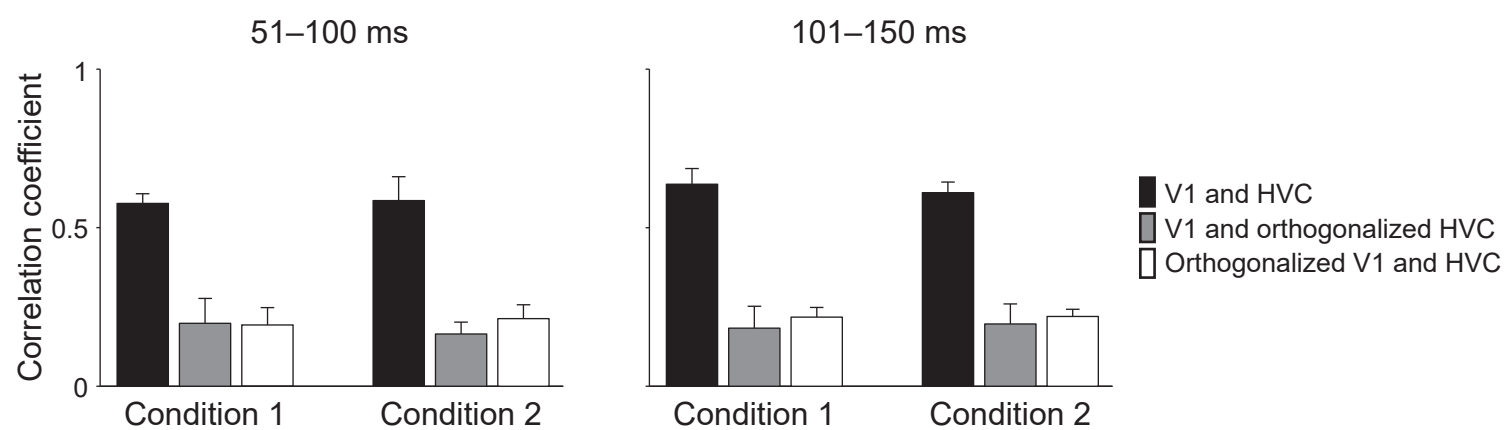

(d)

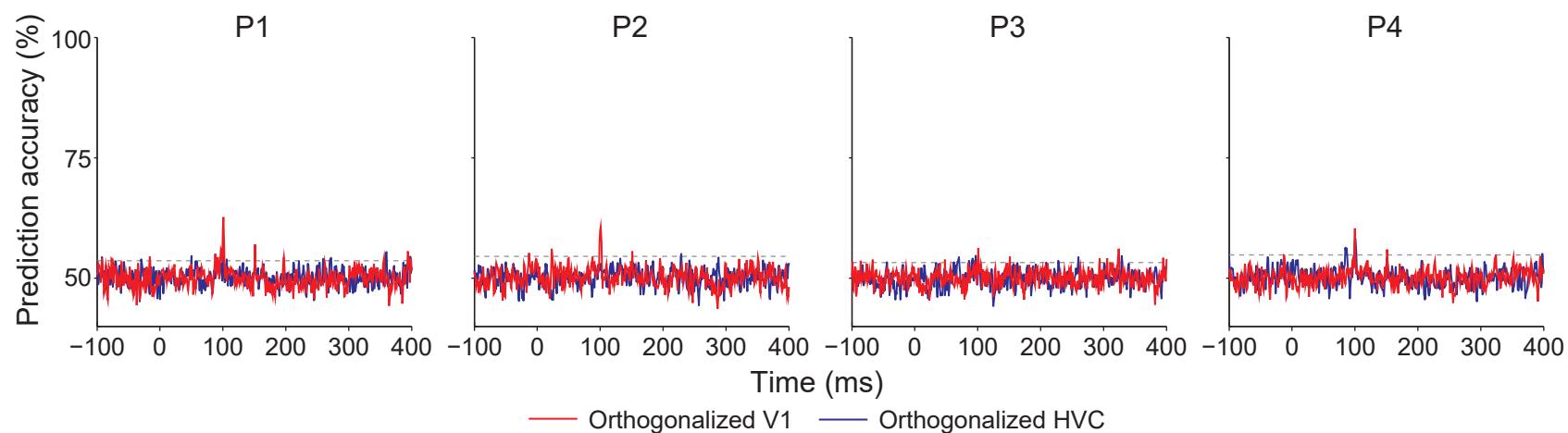

Supplement: S11 Fig — (a) Change of correlation by orthogonalization in simulation data (averaged across participants; error bars, s.d.). (b) Change of prediction accuracy by orthogonalization in simulation data (averaged across participants; error bars, s.d.;). (c) Change of correlation by orthogonalization in real data (averaged across participants; error bars, s.d.). Results of 51–100 ms and 101–150 ms are shown as representative examples. (d) Time courses of prediction accuracy after orthogonalization. Solid lines indicate the prediction accuracy for V1 (red) and HVC (blue) of each participant. Gray dashed lines indicate significance levels. (PDF) [file pone.0198806.s011.pdf]
